# Novel analgesic effects of melanin-concentrating hormone on persistent neuropathic and inflammatory pain in mice

**DOI:** 10.1038/s41598-018-19145-z

**Published:** 2018-01-15

**Authors:** Jae-Hwan Jang, Ji-Yeun Park, Ju-Young Oh, Sun-Jeong Bae, Hyunchul Jang, Songhee Jeon, Jongpil Kim, Hi-Joon Park

**Affiliations:** 1 Acupuncture and Meridian Science Research Center, Kyung Hee University, 26 Kyungheedae-ro, Dongdaemun-gu, Seoul, 02447 Republic of Korea; 2 Department of Korean Medical Science, Graduate School of Korean Medicine, Kyung Hee University, 26 Kyungheedae-ro, Dongdaemoon-gu, Seoul, 02447 Republic of Korea; 3 BK21 PLUS Korean Medicine Science Center, College of Korean Medicine, Kyung Hee University, 26 Kyungheedae-ro, Dongdaemoon-gu, Seoul, 02447 Republic of Korea; 4 College of Korean Medicine, Daejeon University, 62 Daehak-ro, Dong-gu, Daejeon, 34520 Republic of Korea; 5 Mibyeong Research Center, Korean Institute of Oriental Medicine, 1672 Yuseong-daero, Yuseong-gu, Daejeon, 34054 Republic of Korea; 6 Department of Biomedical Sciences, Center for Creative Biomedical Scientists at Chonnam National University, Gwangju, 61469 Republic of Korea; 7 Department of Biomedical Engineering, Dongguk University, Dongguk-Ro 32, Goyang, 10326 Republic of Korea

## Abstract

The melanin-concentrating hormone (MCH) is a peptidergic neuromodulator synthesized by neurons in the lateral hypothalamus and zona incerta. MCHergic neurons project throughout the central nervous system, indicating the involvements of many physiological functions, but the role in pain has yet to be determined. In this study, we found that pMCH^−/−^ mice showed lower baseline pain thresholds to mechanical and thermal stimuli than did pMCH^+/+^ mice, and the time to reach the maximum hyperalgesic response was also significantly earlier in both inflammatory and neuropathic pain. To examine its pharmacological properties, MCH was administered intranasally into mice, and results indicated that MCH treatment significantly increased mechanical and thermal pain thresholds in both pain models. Antagonist challenges with naltrexone (opioid receptor antagonist) and AM251 (cannabinoid 1 receptor antagonist) reversed the analgesic effects of MCH in both pain models, suggesting the involvement of opioid and cannabinoid systems. MCH treatment also increased the expression and activation of CB1R in the medial prefrontal cortex and dorsolateral- and ventrolateral periaqueductal grey. The MCH1R antagonist abolished the effects induced by MCH. This is the first study to suggest novel analgesic actions of MCH, which holds great promise for the application of MCH in the therapy of pain-related diseases.

## Introduction

Pain is a universal experience from birth until the end of life. More than 80% of individuals seeking medical care cite pain as the reason for doing so, citing the significant impact that pain has had on their quality of life. Various pharmacological treatments, such as opiates, are used to relieve chronic pain^1–4^. However, these treatments often cannot meet patients’ needs due to unsatisfactory efficacy and adverse side effects^3,5,6^. Therefore, new analgesic agents without adverse effects are required.

Melanin-concentrating hormone (MCH) is a cyclic 19-amino acid neuropeptide synthesized by neurons in the lateral hypothalamus and zona incerta^7^. The MCH system was originally implicated in energy homeostasis, such as feeding and metabolic activity, but is also involved in the regulation of sleep^8,9^, mood^10,11^, and reward^12,13^. The biological functions of MCH are mediated by two G-protein coupled receptors, the MCH 1 receptor (MCH1R) and the MCH 2 receptor (MCH2R)^14^. MCH1R is widely distributed throughout the rodent^15^ and human brains^16^, whereas MCH2Rs are present in humans but not rodents^17^. MCHergic neurons project throughout the rodent central nervous system (CNS) to areas such as the somatosensory cortex, isocortex, caudate putamen, hippocampus, nucleus accumbens, and amygdala^7,18^, which suggests that the MCH system may participate in a broader spectrum of physiological functions in the CNS than is currently thought. However, the role of MCH in pain modulation has yet to be determined.

Therefore, the present study aimed to elucidate the role of MCH in pain modulation by investigating the anti-nociceptive activities of MCH using pre-MCH (pMCH)^−/−^ and pMCH^+/+^ mice in models of inflammatory pain induced by complete Freund’s adjuvant (CFA) and neuropathic pain induced by partial sciatic nerve ligation (PSNL). Next, to investigate its therapeutic value, the effects of intranasal (i.n.) MCH on inflammatory and neuropathic pain were examined. Finally, the roles of endogenous opioid, cannabinoid, adrenergic, and serotonin receptors in the analgesic effects of MCH were also investigated.

## Results

### pMCH was associated with the modulation of pain responses

**Figure 1:**
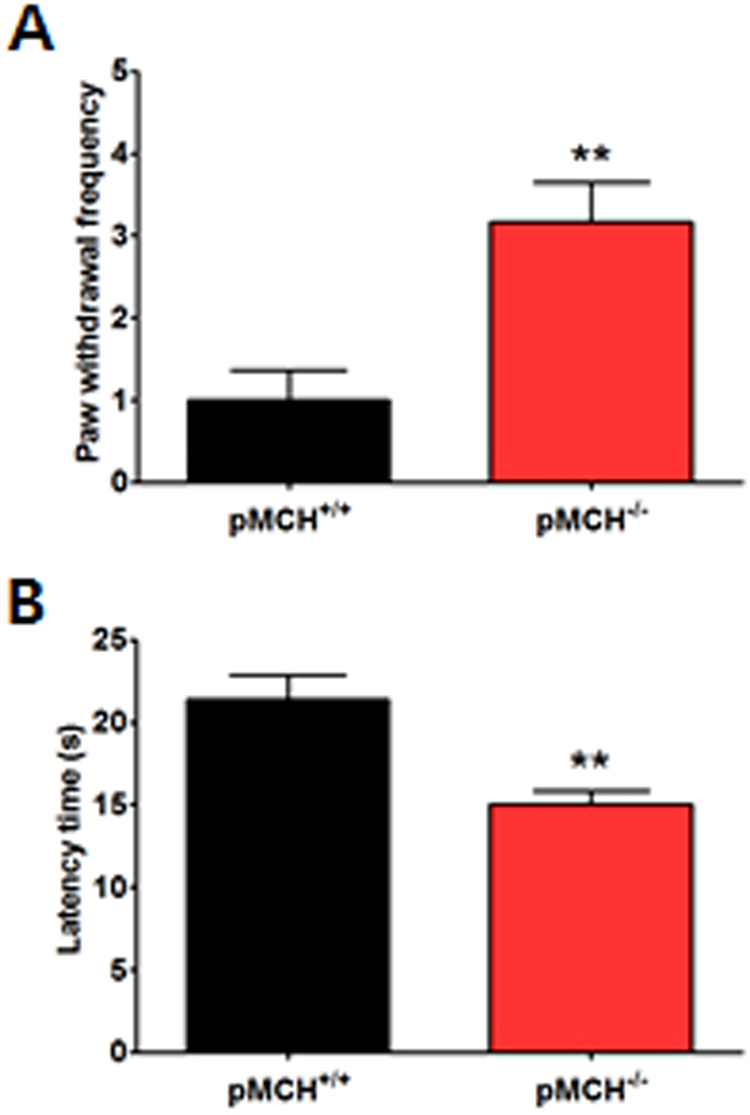
pMCH was associated with the modulation of pain responses. Bar graphs summarizing paw withdrawal frequency in response to mechanical stimuli (**A**) and withdrawal latency time in response to heat stimuli (**B**) in the pMCH^−/−^ and pMCH^+/+^ groups (*n* = 5–6/group). **p < 0.01 compared to the pMCH^+/+^ group, unpaired two-tailed *t-*test. Data are expressed as a mean ± SEM.

To investigate the role of MCH in pain responses, we examined whether the nociceptive behaviors differed between pMCH^−/−^ and pMCH^+/+^ mice using the von Frey and hot-plate tests. Unpaired two-tailed *t*-tests revealed that the withdrawal frequency in pMCH^−/−^ mice was significantly higher than that in pMCH^+/+^ mice (t_10_ = 3.606, *p* = 0.0048; Fig. 1A). In addition, pMCH^−/−^ mice had a significantly shorter latency time than pMCH^+/+^ mice for the withdrawal response to heat stimuli (t_8_ = 3.787, *p* = 0.0053; Fig. 1B).

### Pain responses were increased in pMCH^−/−^ mice compared to pMCH^+/+^ mice in the CFA-induced inflammatory pain model

**Figure 2:**
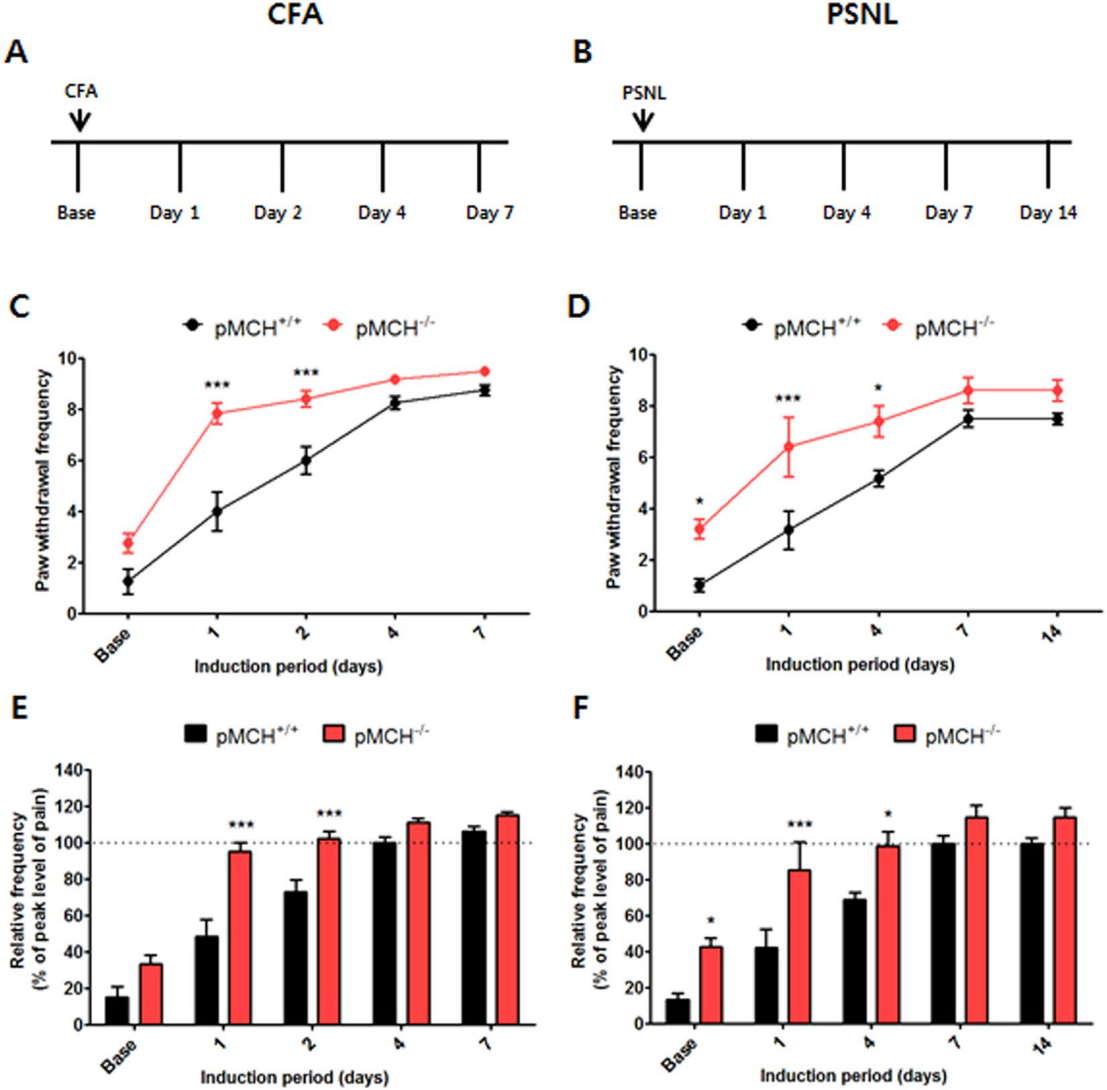
Increased pain response in pMCH^−/−^ mice in the CFA- and PSNL-induced pain models. CFA-induced pain model experiments (**A**,**C**,**E**). PSNL-induced pain model experiments (**B**,**D**,**F**). Experimental designs of the CFA- and PSNL-induced pain model experiments shown in **A** and **B**, respectively. Graphs summarizing paw withdrawal frequency in response to mechanical allodynia (**C**,**D**). Bar graphs summarizing the peak level of pain during mechanical allodynia (**E**,**F**). *n* = 5–6 in all groups. *p < 0.05, ***p < 0.001 compared with the pMCH^+/+^ group. All data were analyzed by two-way RMANOVAs followed by post hoc Bonferroni tests. The results are expressed as means ± SEM.

The CFA-induced pain model has been commonly used to assess chronic inflammatory pain^2^. In the pMCH^+/+^ animals, mechanical allodynia increased gradually after CFA injection and peaked at 4 days after CFA injection, consistent with a previous study^2^. A two-way repeated measures ANOVA (RMANOVA) revealed a significant main effect of genotype (F_1,58_ = 28.85, *p* = 0.0003) and a significant genotype × time interaction (F_4,58_ = 6.062, *p* = 0.0007) for withdrawal frequency. Bonferroni post hoc tests showed that, after CFA injection, mechanical allodynia was significantly greater in pMCH^−/−^ mice than in pMCH^+/+^ mice on Days 1 and 2 (each *p* < 0.001), but there were no differences between the two groups on Days 4 and 7 (*p* > 0.05) because the pain reached almost peak levels at these time points (Fig. 2A and C).

To determine whether there were any differences in the induction time to reach peak levels of pain, the withdrawal frequency was converted to the relative value of the peak level of pain, and the data were compared. The analysis of relative frequency with a two-way RMANOVA revealed a significant main effect of genotype (F_1,58_ = 28.86, *p* = 0.0003), which indicates that the knockout (KO) mice exhibited a significantly greater degree of pain than the wild-type (WT) mice. There was also a significant genotype × time interaction (F_4,58_ = 6.052, *p* = 0.0007), which indicates that there was a significant difference in the increase in pain between the two groups. Bonferroni post hoc tests showed that the peak allodynic behavior occurred significantly earlier in pMCH^−/−^ mice than in pMCH^+/+^ mice on Days 1 and 2 after the injection (each *p* < 0.001 vs. pMCH^+/+^). After reaching peak levels of pain, the withdrawal frequency was still higher in pMCH^−/−^ mice (111% and 109% of pMCH^+/+^ mice on Days 4 and 7, respectively), but these differences did not reach statistical significance (each *p* > 0.05; Fig. 2E).

### Pain responses were increased in pMCH^−/−^ mice compared to pMCH^+/+^ mice in the PSNL-induced neuropathic pain model

We also examined the role of the pMCH gene in the PSNL-induced neuropathic pain model using pMCH^−/−^ and pMCH^+/+^ mice. In the PSNL-induced pain model, mice gradually developed mechanical allodynia over the 7 days after PSNL surgery, with the peak level occurring at day 7, consistent with previous studies^19,20^. A two-way RMANOVA revealed a significant main effect of genotype (F_1,53_ = 12.32, *p* = 0.0080) and a significant genotype × time interaction (F_4,53_ = 3.181, *p* = 0.0262) for the withdrawal frequency in PSNL mice. Similar to the results in the CFA-induced pain model, Bonferroni post hoc tests showed that mechanical allodynia was significantly greater in pMCH^−/−^ mice than in pMCH^+/+^ mice from Days 1 to 4 after surgery (each *p* < 0.05 on Days 1 and 4), but it did not significantly differ on Days 7 and 14 (Fig. 2B and D).

A two-way RMANOVA assessing the relative frequency of the peak level of pain revealed a significant main effect of genotype (F_1,53_ = 12.34, *p* = 0.0079), which indicates that the KO mice exhibited a significantly greater degree of pain than the WT subjects. There was also a genotype × time interaction (F_4,53_ = 3.180, *p* = 0.0262), which indicates that there was a significant difference between the two groups in the increase in pain. Bonferroni post hoc tests showed that the peak levels of pain occurred significantly earlier after PSNL surgery in pMCH^−/−^ mice than in pMCH^+/+^ mice (*p* < 0.001 on Day 1 and *p* < 0.05 on Day 4). After peak levels of pain were reached, the withdrawal frequency was still higher in pMCH^−/−^ mice (113% of pMCH^+/+^ mice on both Days 7 and 14), but these differences were not significant (*p* > 0.05; Fig. 2F).

### I.n. MCH treatment increased the thermal pain threshold in a dose-dependent manner before exploring the therapeutic effect of MCH in pain models

**Figure 3:**
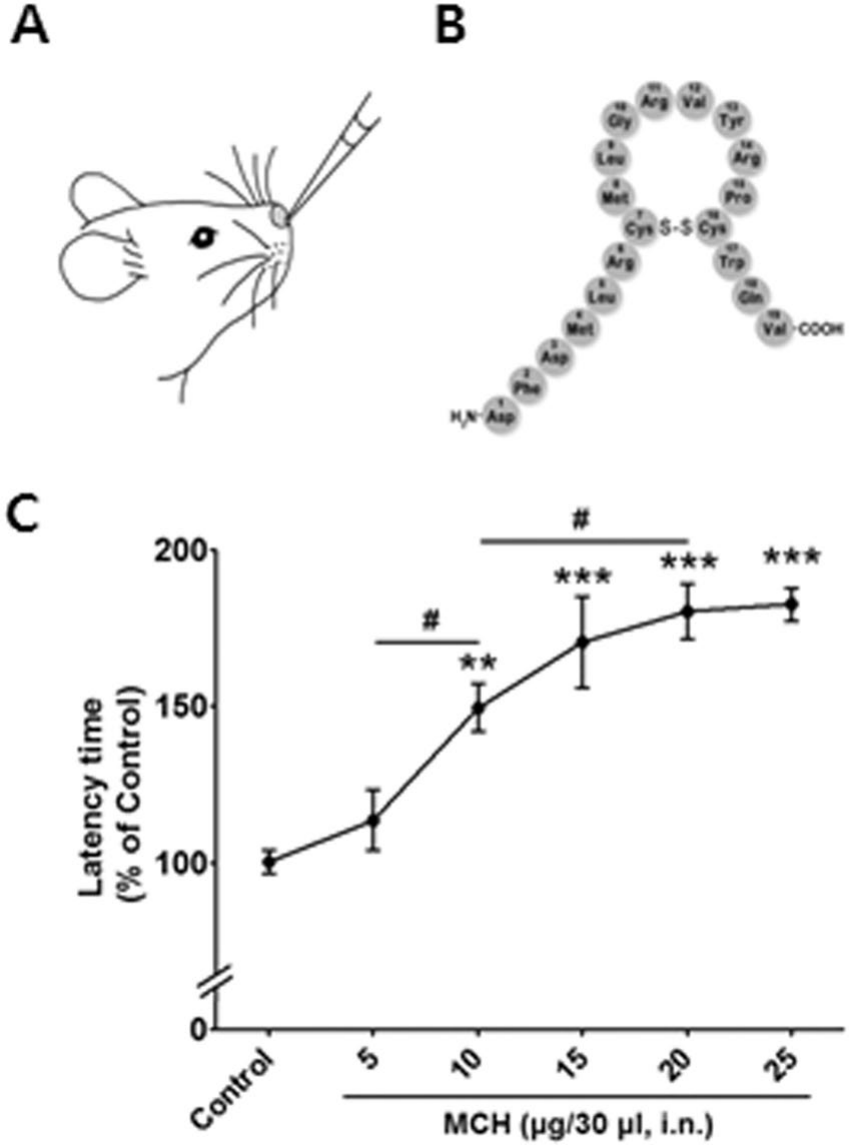
Antinociceptive effects of i.n. MCH in the hot-plate test. I.n. MCH treatment in a mouse (**A**). Chemical structure of MCH (**B**). The hot-plate test was performed 30 min after MCH treatment (5, 10, 15, 20, and 25 μg/30 μl, i.n.) or saline (30 μl, i.n.) administration (**C**). n = 6/group. **p < 0.01, ***p < 0.001 compared to Control group. ^#^ *p* < 0.05 compared to the other dose group. The data were analyzed with a one-way ANOVA followed by Newman–Keuls post hoc tests. The results are expressed as means ± SEM. i.n.: intranasal.

We examined the effective dose range of MCH to accurately evaluate the antinociceptive actions of MCH using the hot-plate test. Latency time was measured 30 min after either MCH treatment (5–25 μg/30 μl, i.n.) or saline administration (i.n.); the changes in antinociceptive behaviors induced by different MCH doses are shown in Fig. 3. A one-way ANOVA revealed a significant difference between groups (F_5,30_ = 15.21, *p* < 0.0001), and Newman–Keuls post hoc tests showed that treatment with MCH increased the thermal threshold in a dose-dependent manner such that a significant difference was observed with a 10 μg/30 μl MCH dose (*p* < 0.01 vs. Control; *p* < 0.05 vs. 5 μg/30 μl MCH). The maximum antinociceptive effect of MCH estimated from the dose–response curve was 20 μg/30 μl (*p* < 0.001 vs. Control; *p* < 0.05 vs. 10 μg/30 μl MCH; Fig. 3C).

### Effects of a single i.n. MCH treatment in the CFA-induced inflammatory pain model

**Figure 4:**
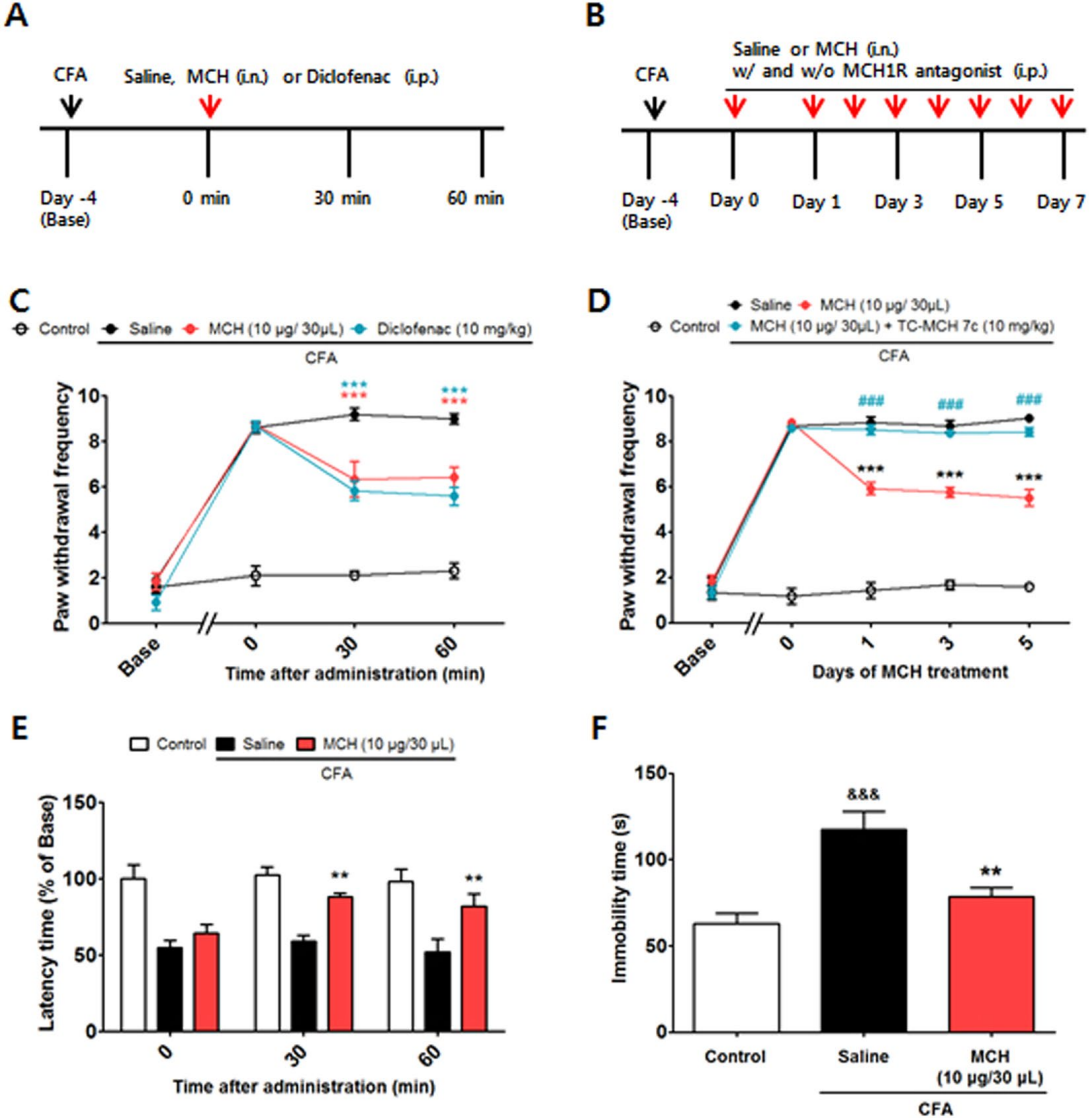
Analgesic effects of i.n. MCH on CFA-evoked inflammatory pain and the MCH1R-induced mediation of these effects. Experimental design of the single (**A**) and repeated (**B**) treatments of MCH in the CFA-induced pain model. The anti-allodynic and analgesic effects of MCH were measured 30 and 60 min after saline, respectively. Administration of either MCH (10 μg/30 μl, i.n.) or diclofenac (10 mg/kg) in the von Frey test (**C**) and hot-plate test (**E**). A MCH1R antagonist, TC-MCH 7c (10 mg/kg/day for 5 days, i.p.), was injected 30 min before MCH (10 μg/30 μl/day for 5 days, i.n.) or saline treatment, and the von Frey test was performed 30 min after MCH treatment (**D**). *n* = 5 − 6/group. *p < 0.05, **p < 0.01, and ***p < 0.001 compared with the CFA group and ^###^ *p* < 0.001 compared with the MCH group. Data were analyzed with a two-way RMANOVA followed by post hoc Bonferroni tests. The TST was performed 12 days after CFA or saline injection (7^th^ day of MCH or saline treatment; **F**). *n* = 5/group. **p < 0.01 vs. the CFA group, ^&&&^p < 0.001 vs. the Control group. The data were analyzed with a one-way ANOVA followed by Newman–Keuls post hoc tests. Data are expressed as means ± SEM. i.n.: intranasal, i.p.: intraperitoneal.

The anti-allodynic and analgesic effects of MCH were measured using the von Frey and hot-plate tests in the CFA-induced inflammatory pain model. The baseline measurement was performed 1 day before CFA injection and either MCH (10 μg/30 μl, i.n.) or saline (i.n.) was administered on Day 4 after the CFA injection (Fig. 4A). For mechanical allodynia, a two-way RMANOVA revealed a significant main effect of group (F_3,106_ = 104.4, *p* < 0.0001) and a significant group × time interaction (F_9,106_ = 18.56, *p* < 0.0001). Bonferroni post hoc tests showed significant improvements in the MCH group compared to the CFA group (each *p* < 0.001 vs. CFA). The analgesic effects of MCH were compared with those of a non-steroidal anti-inflammatory drug (NSAID), diclofenac (10 mg/kg, intraperitoneal [i.p.]); MCH (10 μg/30 μl, i.n.) produced the same level of analgesia as diclofenac (diclofenac vs. CFA, *p* < 0.001: diclofenac vs. MCH, *p* > 0.05; Fig. 4C).

In terms of thermal hyperalgesia, a two-way RMANOVA revealed a significant main effect of group (F_2,45_ = 20.57, *p* < 0.0001) and a significant group × time interaction (F_4,45_ = 1.721, *p* = 0.1755). Bonferroni post hoc tests showed significant improvements in latency time in the MCH group (*p* < 0.01 vs. CFA; Fig. 4E).

### Effects of repeated i.n. MCH treatments in the CFA-induced pain model and the involvement of MCH1R

The effects of repeated treatments with MCH (10 μg/30 μl, i.n.) over 7 days in the CFA-induced inflammatory pain model were also investigated in the present study (Fig. 4B). Additionally, we examined whether the effects elicited by MCH treatment were specifically associated with MCH1R following pretreatment with a MCH1R antagonist (TC-MCH 7c, 10 mg/kg, i.p.). A two-way RMANOVA revealed a significant main effect of group (F_3,116_ = 363.4, *p* < 0.0001) and a significant group × day interaction (F_12,116_ = 55.50, *p* < 0.0001) for mechanical allodynia measured over 7 days. Bonferroni post hoc tests showed that the anti-allodynic effects of repeated MCH treatments were significant compared to those in the CFA group (*p* < 0.001) and were significantly abolished by the MCH1R antagonist (*p* < 0.001 vs. MCH group; Fig. 4D).

Several studies have demonstrated that chronic pain produces long-term sensitization and generates depression-like behaviors^21–23^. Thus, the present study evaluated the anti-depressant effects of MCH (10 μg/30 μl/day for 7 days, i.n.) on depression-like behaviors generated in this pain model using the tail suspension test (TST). A one-way ANOVA (F_2,12_ = 14.01, *p* > 0.0001) followed by Newman–Keuls post hoc tests revealed a significant increase in immobility time in the CFA group (*p* < 0.001 vs. Control), whereas MCH treatment significantly attenuated immobility time (*p* < 0.01 vs. CFA; Fig. 4F).

### Effects of i.n. MCH treatment in the mouse PSNL-induced neuropathic pain model

**Figure 5:**
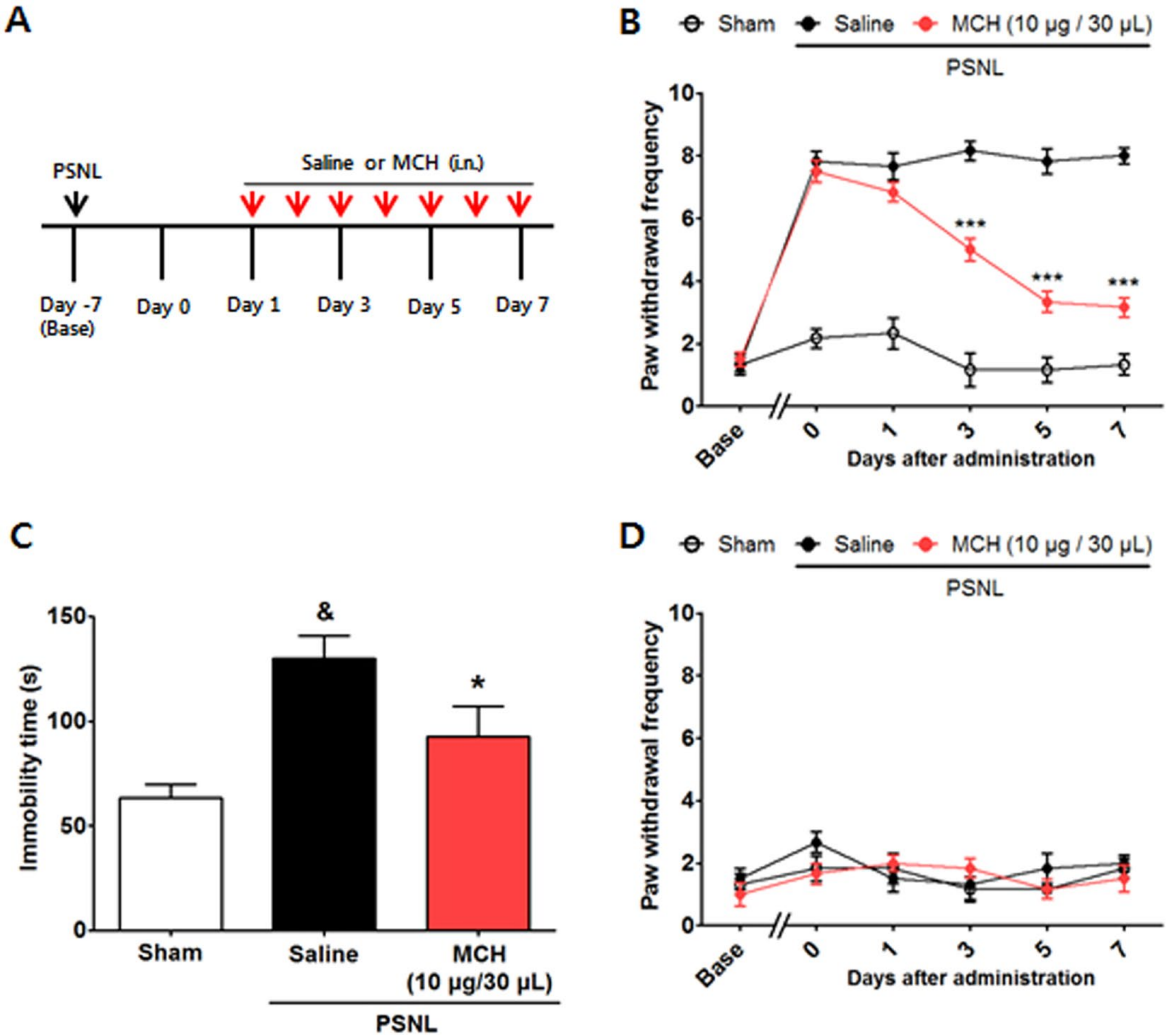
Analgesic effects of i.n. MCH on PSNL-induced neuropathic pain. Experimental design of the PSNL-induced pain model experiments (**A**). Anti-allodynic effects of MCH measured on the ipsilateral (**B**) and contralateral (**D**) plantar surfaces 30 min after the 7^th^ administration of MCH (10 μg/30 μl/day for 7 days i.n., each *n* = 6) according to the von Frey test. ***p < 0.001 vs. the PSNL group. Data were analyzed with a two-way RMANOVA followed by post hoc Bonferroni tests. The FST was performed 14 days after the sham or PSNL surgery (7^th^ day of MCH or saline treatment; **C**). Control group: *n* = 3, all other groups: *n* = 5–7. *p < 0.05 vs. CFA group, ^&^ *p* < 0.05 vs. Sham. The data were analyzed with a one-way ANOVA followed by Newman–Keuls post hoc tests. Data are expressed as means ± SEM. i.n.: intranasal.

To examine the effects of MCH treatment (10 μg/30 μl/day, i.n.) on PSNL-induced hypersensitivity (Fig. 5A), we began to administer drugs to the mice 7 days after surgery when the mechanical allodynia was significantly induced; this treatment was continued for 7 consecutive days. A two-way RMANOVA revealed a significant main effect of group (F_2,105_ = 360.5, *p* < 0.0001) and a significant group × day interaction (F_10,105_ = 20.23, *p* < 0.0001). Bonferroni post hoc tests showed that MCH reversed the established ipsilateral mechanical allodynia at each time point (each *p* < 0.001 vs. PSNL on Days 3, 5, and 7 after treatment; Fig. 5B). There were no significant changes in the pain levels of the contralateral hind paws (Fig. 5D).

The present study also evaluated the influence of MCH on depression-like symptoms in this pain model using the forced swim test (FST). A one-way ANOVA (F_2,12_ = 5.199, *p* > 0.0001) followed by Newman–Keuls post hoc tests showed that there was a significant increase in immobility time in the PSNL group (*p* < 0.05 vs. Sham), whereas MCH treatment significantly attenuated this behavior (*p* < 0.05 vs. PSNL; Fig. 5C).

### The anti-allodynic and analgesic effects of MCH were fully blocked by naltrexone and AM251

**Figure 6:**
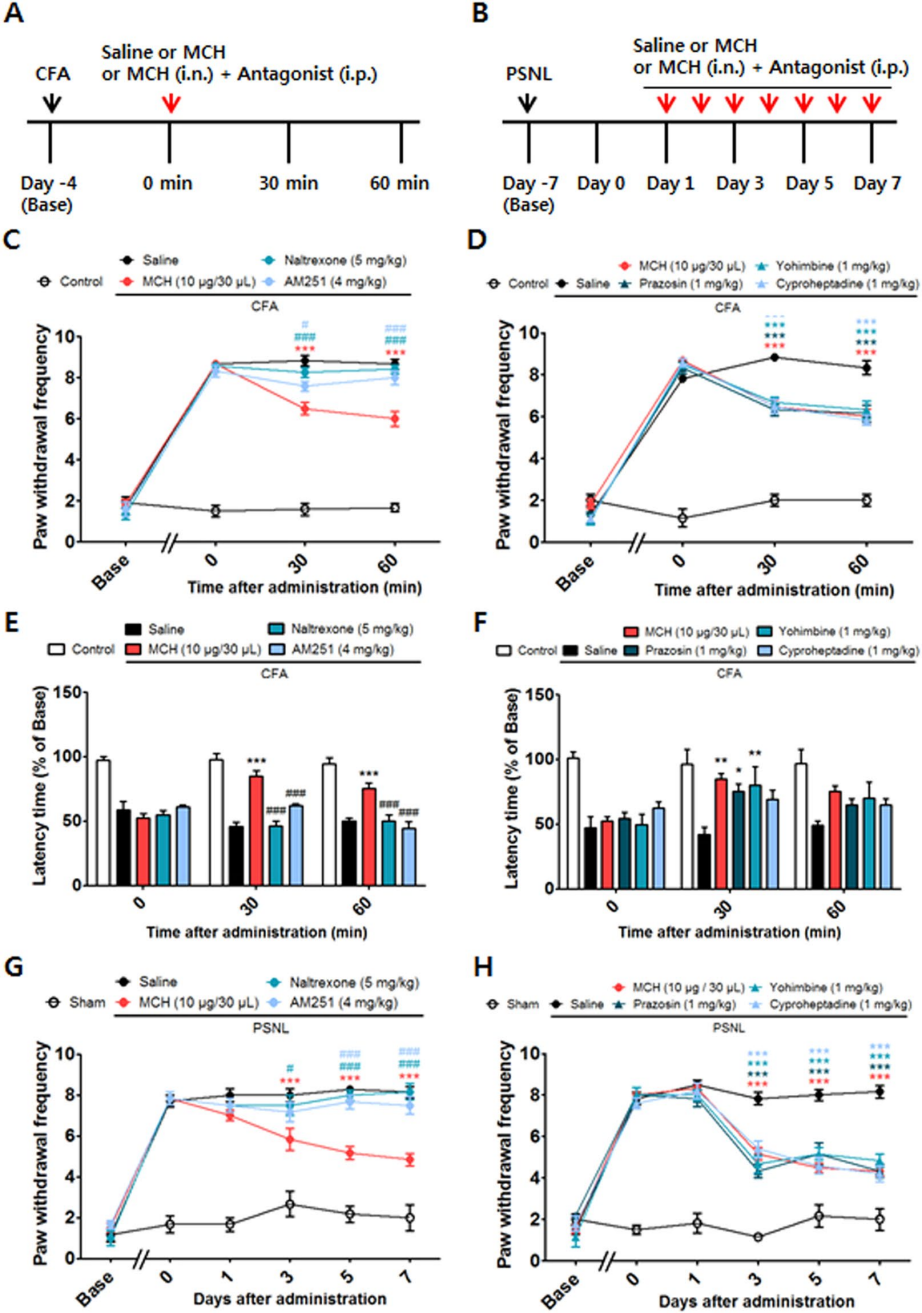
Effects of opioid and cannabinoid receptor antagonists on the analgesic effects of i.n. MCH following CFA-induced inflammatory pain and PSNL-evoked neuropathic pain. Experimental design of CFA (**A**) and PSNL (**B**). Opioid (naltrexone, 5 mg/kg, i.p.) and CB1R (AM251, 4 mg/kg, i.p.) antagonist treatments (**C**,**E**, and **G**) and α1 adrenergic (prazosin, 1 mg/kg; i.p), α2 adrenergic (yohimbine, 1 mg/kg, i.p.), and 5-HT1/2 (cyproheptadine, 1 mg/kg, i.p.) receptor antagonists (**D**,**F**, and **H**) were administered 30 min prior to treatment with either MCH (10 μg/30 μl, i.n.) or saline. The behavioral tests were performed 30 and 60 min after MCH or saline treatment in the CFA-induced inflammatory pain model (**C**,**D**,**E**, and **F**; *n* = 6–7/group). In the PSNL-evoked neuropathic pain model, the behavioral tests were performed 30 min after saline or MCH (10 μg/30 μl/day for 7 days, i.n.) treatment (**G**,**H**; *n* = 6/group). The von Frey test (**C**,**D**,**G**, and **H**) and hot-plate test (**E**,**F**). ***p < 0.001 vs. the CFA or PSNL groups. ^#^ *p* < 0.05, ^##^ *p* < 0.01, ^###^ *p* < 0.001 compared with the MCH (10 μg/30 μl) group. All data were analyzed with a two-way RMANOVA followed by post hoc Bonferroni tests. The results are expressed as means ± SEM. i.n.: intranasal, i.p.: intraperitoneal.

To examine the involvement of the opioid, cannabinoid, adrenergic, and serotonin systems, an opioid receptor antagonist (naltrexone, 5 mg/kg, i.p.), a cannabinoid 1 receptor (CB1R) antagonist (AM251, 4 mg/kg, i.p.), an α1 adrenoreceptor antagonist (prazosin hydrochloride, 1 mg/kg, i.p.), an α2 adrenoreceptor antagonist (yohimbine hydrochloride, 1 mg/kg, i.p.), and a 5-HT1/2 receptor antagonist (cyproheptadine hydrochloride, 1 mg/kg, i.p.) were independently administered 30 min prior to MCH treatment (10 μg/30 μl, i.n.) in the inflammatory pain model (Fig. 6A). For mechanical allodynia, a two-way RMANOVA revealed a significant main effect of group (F_4,115_ = 118.0, *p* < 0.0001) and a significant group × time interaction (F_12,115_ = 65.57, *p* < 0.0001). Bonferroni post hoc tests showed that the anti-allodynic effects of MCH were independently and significantly blocked by naltrexone and AM251 (each *p* < 0.001 vs. MCH; Fig. 6C). For thermal hyperalgesia, a two-way RMANOVA revealed a significant main effect of group (F_4,85_ = 57.05, *p* < 0.0001) and a significant group × time interaction (F_8,85_ = 7.954, *p* < 0.0001). Bonferroni post hoc tests showed that the anti-allodynic effects of MCH were independently and significantly blocked by naltrexone and AM251 (each *p* < 0.001 vs. MCH; Fig. 6E). On the other hand, α1 adrenergic, α2 adrenergic, and 5-HT1/2 receptor antagonists did not reduce these effects (Fig. 6D and F).

The roles that the opioid, cannabinoid, adrenergic, and serotonergic systems play in the effects of MCH (10 μg/30 μl/day for 7 days, i.n.) were also explored in the PSNL-induced neuropathic pain model (Fig. 6B). A two-way RMANOVA revealed a significant main effect of group (F_4,175_ = 85.70, *p* < 0.0001) and a significant group × time interaction (F_20,175_ = 13.77, *p* < 0.0001). Bonferroni post hoc tests showed that the anti-allodynic effects of MCH treatment were independently and significantly blocked by naltrexone and AM251 (each *p* < 0.001 vs. MCH; Fig. 6G) in this neuropathic pain model. On the other hand, α1 adrenergic, α2 adrenergic, and 5-HT1/2 receptor antagonists did not reverse the analgesic effects of MCH (Fig. 6H).

### I.n. MCH activated CB1R in the medial prefrontal cortex

**Figure 7:**
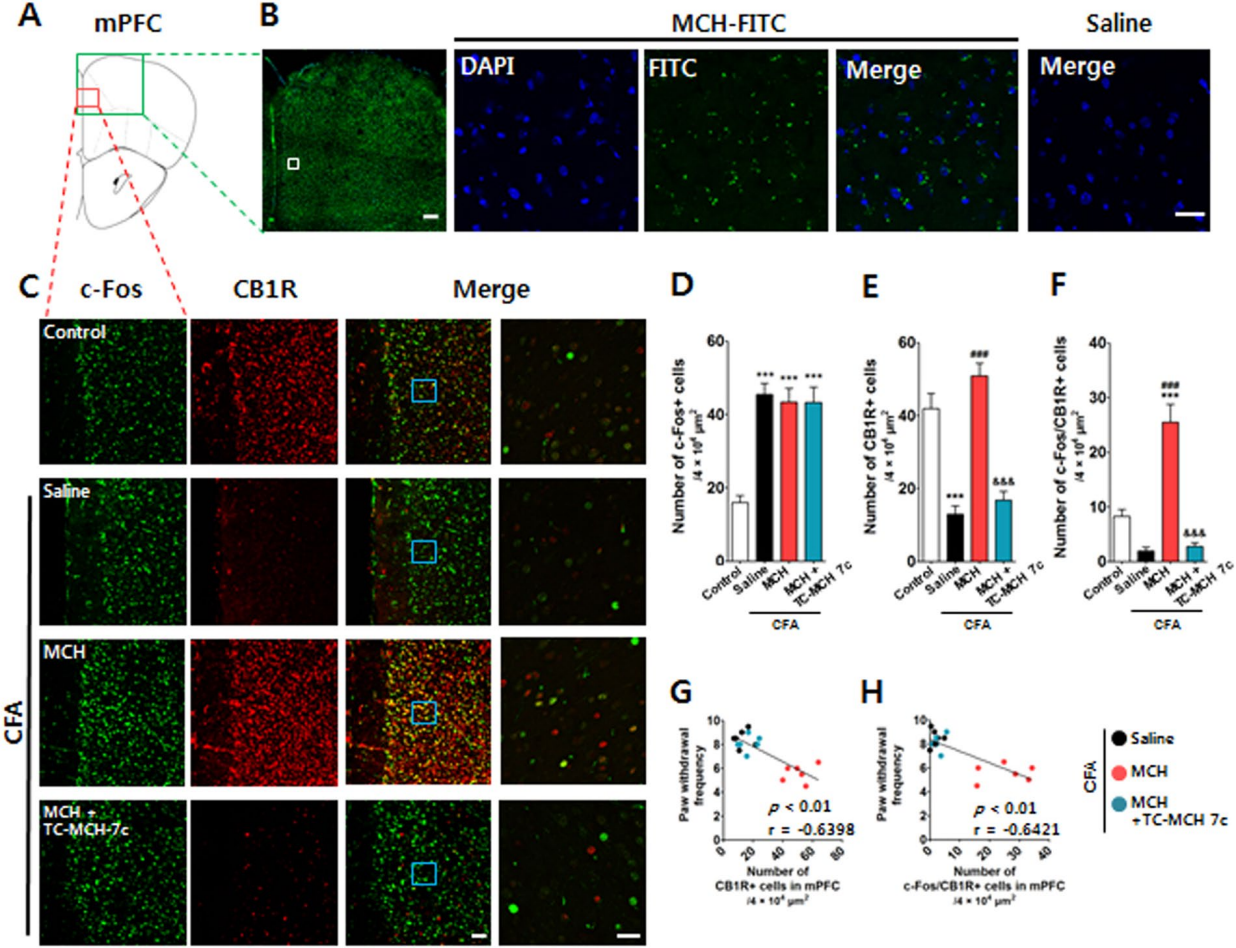
Changes in CB1R in the mPFC after i.n. administration of MCH in the CFA-induced inflammatory pain model. Representative figure showing the mPFC region in the mouse brain (**A**) and the distribution of MCH in the mPFC 30 min after the i.n. administration of MCH-FITC (10 μg/30 μl) or saline. DAPI: blue; MCH-FITC: green. Scale bar: 400 μm and 30 μm (**B**). Representative graphs showing the expression levels and activation of CB1R in the mPFC 2 h after the i.n. administration of MCH (10 μg/30 μl) on the 4^th^ day after CFA injection. A MCH1R antagonist, TC-MCH 7c (10 mg/kg, i.p.), was administered 30 min prior to treatment with MCH. CB1R- and/or c-Fos-positive cells in the mPFC were counted. Scale bar: 100 and 30 μm (**C**). The results were analyzed with a one-way ANOVA followed by Newman–Keuls post hoc tests (**D**–**F**). *n* = 6/group. ***p < 0.001 vs. the Control group, ^###^ *p* < 0.001 vs. the CFA group, and ^&&&^ *p* < 0.001 vs. MCH group. Data are expressed as means ± SEM. Figures show data for the correlation between mechanical allodynia and a change in CB1R after i.n. MCH administration. The r-values were analyzed using a Spearman rank correlation coefficient. mPFC: medial prefrontal cortex; CB1R: cannabinoid 1 receptor; MCH1R: MCH 1 receptor.

Many studies have shown that the medial prefrontal cortex (mPFC) plays important roles in the modulation of the emotional and sensory components of pain^24–26^. To elucidate the involvement of the mPFC in the analgesic effects of MCH, we first used fluorescence imaging with fluorescein (FITC)-labeled MCH (FITC-MCH) applied via the nose (10 μg/30 μl, i.n.) to examine whether intranasally administered MCH was distributed around the mPFC region. Fluorescence histological imaging analyses confirmed that FITC-MCH was located in the regions around the mPFC at 30 min after its i.n. application (Fig. 7A and B).

All CB1R-positive cells were co-stained with NeuN, which suggests that CB1Rs are expressed in neurons (Supplementary Fig. 1). The localization, expression levels, and activation of CB1R within the mPFC regions in mice with CFA-induced pain were also assessed with double immunofluorescence staining of c-Fos (green) and CB1R (red). Preabsorption with corresponding peptide abolished the immunoreactivity, and did control staining when the primary antibody was removed (Supplementary Fig. 3). A one-way ANOVA revealed a significant main effect of group for CB1R expression (F_3,20_ = 33.97, *p* < 0.0001), and Newman–Keuls post hoc tests showed that the expression levels of CB1R were lower in the CFA group than in the saline-treated Control group (*p* < 0.001). Although MCH treatment significantly increased the expression levels of CB1R compared to the saline-treated CFA group (*p* < 0.001), these MCH-induced changes were blocked by treatment with TC-MCH 7c (*p* < 0.001). A one-way ANOVA revealed a significant main effect of group for the double-staining of c-Fos and CB1R (F_3,20_ = 35.79, *p* < 0.0001), and Newman–Keuls post hoc tests showed that the co-expression levels of CB1R and c-Fos in the mPFC region were higher in the MCH group than in the Control or CFA groups (each *p* < 0.001); however, the effects of MCH were inhibited by treatment with TC-MCH 7c (*p* < 0.001; Fig. 7C–F). Additionally, Spearman rank correlation coefficient analyses were performed to investigate whether the changes in CB1Rs reflected changes in pain status. The changes in paw withdrawal frequency were significantly correlated with the increased expression levels and activation of CB1R in the mPFC (r = −0.6398 and *p* < 0.01 for CB1R; r = −0.6421 and *p* < 0.01 for c-Fos/CB1R; Fig. 7G and H).

### I.n. MCH activated CB1R in the ventrolateral periaqueductal gray matter and dorsolateral periaqueductal gray matter

Using double immunofluorescence staining of c-Fos (green) and CB1R (red), we also measured the localization, expression levels, and activation of CB1R within the ventrolateral periaqueductal gray (vlPAG) and dorsolateral PAG (dlPAG), which are important components of the descending inhibitory pain pathway^27,28^, in mice with CFA-induced pain. A one-way ANOVA revealed a significant difference between groups (10 μg/30 μl) for CB1R expression (vlPAG: F_3,20_ = 48.35, *p* < 0.0001; dlPAG: F_3,20_ = 71.22, *p* < 0.0001), and Newman–Keuls post hoc tests showed that the expression levels of CB1R were reduced by the CFA injection (*p* < 0.001 vs. Control). MCH treatment increased the expression levels of CB1R (each *p* < 0.001 vs. saline-treated CFA), but these effects were inhibited by treatment with TC-MCH 7c (10 mg/kg, i.p.; each *p* < 0.001 vs. MCH). A one-way ANOVA revealed a significant difference between groups for the co-expression of c-Fos and CB1R (vlPAG: F_3,20_ = 51.63, *p* < 0.0001; dlPAG: F_3,20_ = 59.73, *p* < 0.0001), and Newman–Keuls post hoc tests showed that the co-expression levels of c-Fos and CB1R were higher in the MCH group than in the Control and CFA groups for both the vlPAG (each *p* < 0.001) and dlPAG (each *p* < 0.001); however, this effect was blocked by treatment with TC-MCH 7c (*p* < 0.001 vs. MCH).

**Figure 8:**
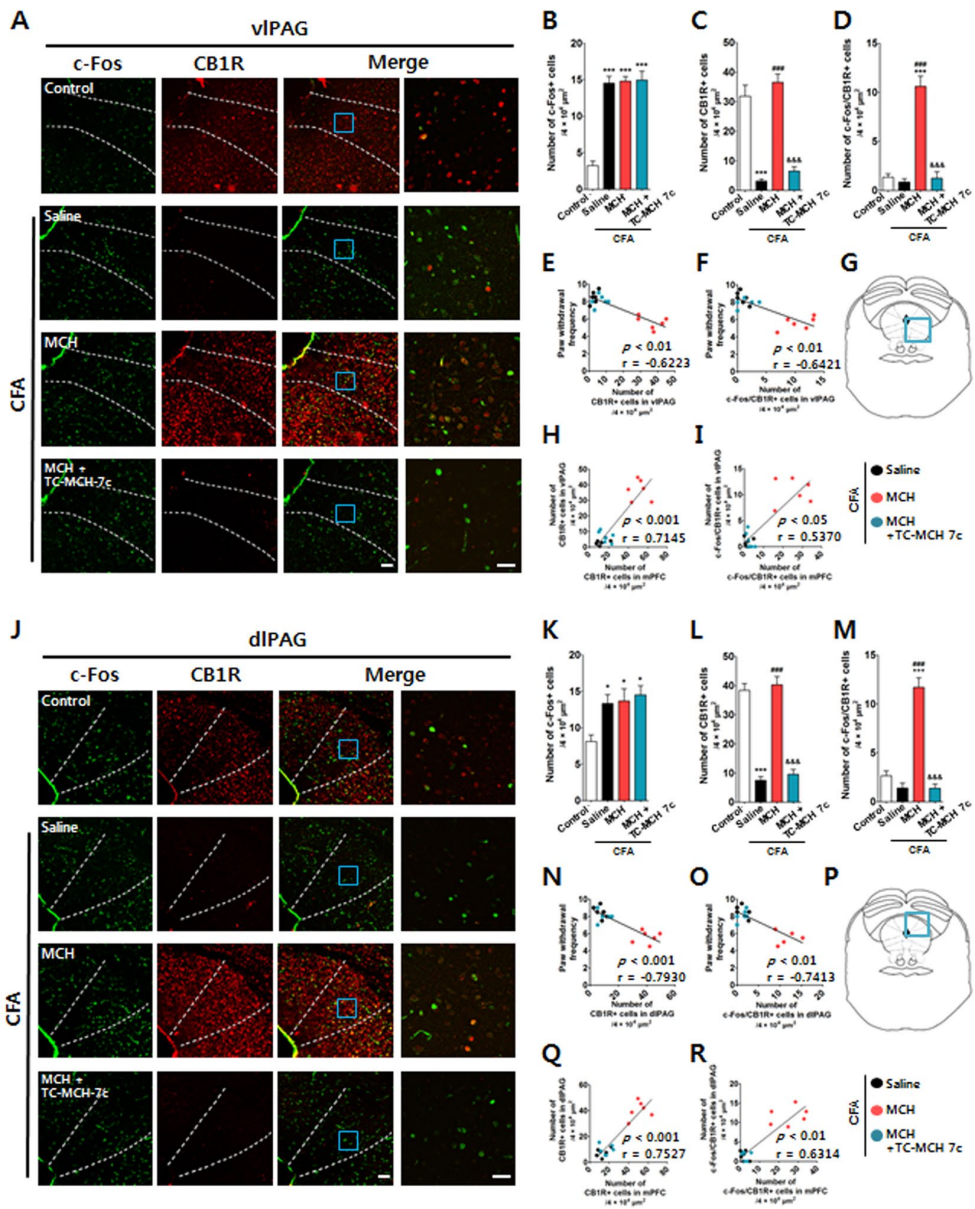
Changes in CB1R in the vlPAG and dlPAG after i.n. administration of MCH in the CFA-induced inflammatory pain model. Histological examination of immunofluorescent tissue sections from the vlPAG (**A**–**I**) and dlPAG (**J**–**R**) of mice showing the activation of CB1R in the vlPAG and dlPAG 2 h after the i.n. administration of MCH (10 μg/30 μl) on the 4^th^ day after CFA injection. A MCH1R antagonist, TC-MCH 7c (10 mg/kg, i.p.), was administered 30 min prior to treatment with MCH. Cells positive for CB1R (red) and/or c-Fos (green) in the vlPAG (**A**) and dlPAG (**J**) were visualized under a microscope. CB1R- and/or c-Fos-positive cells in the vlPAG (**B**–**D**) and dlPAG (**K**–**M**) were counted. PAG structure: vlPAG region (**G**) and dlPAG region (**P**). Scale bar: 100 and 30 μm. All groups: *n* = 6. *p < 0.05, ***p < 0.001 vs. the Control group, ^###^ *p* < 0.001 vs. the CFA group, and ^&&&^ *p* < 0.001 vs. MCH group. The data were analyzed with a one-way ANOVA followed by Newman–Keuls post hoc tests (**B**–**D** and **G**–**I**). Data are expressed as means ± SEM. The expression levels and activation of CB1R in the vlPAG and dlPAG, respectively, were correlated with paw withdrawal frequency (**E**,**F**,**N**, and **O**) or with those in the mPFC (**H**,**I**,**Q**, and **R**). The r-values were analyzed with the Spearman rank correlation coefficient. vlPAG: ventrolateral PAG; dlPAG: dorsolateral PAG; CB1R: cannabinoid 1 receptor.

Furthermore, the expression levels and activation of CB1R exhibited interactions with paw withdrawal frequency and the mPFC, respectively, following CFA-induced pain. A Spearman rank correlation coefficient analysis showed that reductions in paw withdrawal frequency were correlated with the increased expression levels and activation of CB1R in the vlPAG (r = −0.6223, *p* < 0.01 for CB1R; r = −0.6421, *p* < 0.01 for c-Fos/CB1R) and the dlPAG (r = −0.7930, *p* < 0.001 for CB1R; r = −0.7413, *p* < 0.01 for c-Fos/CB1R). The Spearman rank correlation analysis also revealed that the increased expression levels and activation of CB1R in the mPFC interacted with those in the vlPAG (r = 0.7145, *p* < 0.001 for CB1R; r = 0.5370, *p* < 0.05 for c-Fos/CB1R) and dlPAG (r = 0.7527, *p* < 0.001 for CB1R; r = 0.6314, *p* < 0.01 for c-Fos/CB1R; Fig. 8).

## Discussion

Chronic pain affects millions of individuals around the world and is often accompanied by dramatic changes in quality of life. The needs of patients with chronic pain are largely unmet due to the poor efficacy and side effects of current treatments, and therefore, the development of new treatments is necessary. This study indicated that the hypothalamic neuropeptide MCH mediates the development of pain behaviors in pMCH^−/−^ mice. Although the i.n. administration of MCH inhibited nociceptive behaviors associated with CFA-induced inflammatory pain, these MCH-induced effects were blocked by a MCH1R antagonist. The effects of MCH were also evident in the PSNL-induced neuropathic pain model. Furthermore, the present study demonstrated the possible roles of the endogenous opioid and endocannabinoid analgesic systems in the antinociceptive effects of MCH. I.n. treatment with MCH increased the number of CB1Rs as well as the activation of this system, and these changes were negatively correlated with the pain behaviors in the mPFC, vlPAG, and dlPAG. To our knowledge, this is the first report suggesting an analgesic effect of MCH in pain models.

MCH is synthesized exclusively in the lateral hypothalamus and zona incerta but MCH neurons project widely throughout the CNS, which suggests that MCH may function as a neurotransmitter and/or neuromodulator to regulate many physiological functions, including sleep, feeding, and reward^12,29–31^. While the role of MCH in pain was not revealed until recently, microdialysis studies showed that MCH concentrations in the amygdala are relatively lower in epileptic patients upon feeling pain and that these levels are restored by morphine treatment^32^. The present study aimed to determine the involvement of MCH in pain modulation by comparing nociceptive behaviors between pMCH^−/−^ and pMCH^+/+^ mice. The results indicated that the baseline pain sensitivity was greater in pMCH^−/−^ than pMCH^+/+^ mice. The time to reach the maximum level of mechanical allodynia was significantly shorter in pMCH^−/−^ mice than pMCH^+/+^ controls in both the CFA-induced inflammatory and PSNL-induced neuropathic pain models. In the later stages of pain (after Days 4 and 7 of pain induction in each model), the levels of pain remained higher in pMCH^−/−^ mice than in pMCH^+/+^ mice, but these differences were not significant. It is possible that the pain response could not increase further after reaching a peak level. Taken together, the present results indicate that MCH plays important roles in the modulation of pain. However, pMCH encodes MCH as well as two neuropeptides, neuropeptide EI (NEI) and neuropeptide GE (NGE)^33^, and may cause an alternative splice variant, MCH-gene-overprinted-polypeptide (MGOP), and an encode antisense-RNA-overlapping-MCH (AROM)^34,35^. Therefore, MCH as well as other proteins encoded by pMCH, including NEI, NGE, MGOP, and AROM, may contribute to the phenotype of pMCH^−/−^ mice.

Next, to investigate the role of MCH in the modulation of pain, we tested whether the i.n. administration of MCH could modulate pain behaviors via neurological activity. As expected, administration of MCH (10–25 μg/30 μl, i.n.) exerted dose-dependent antinociceptive effects against thermal noxious stimuli in the hot-plate test.

Additionally, both a single treatment (10 μg/30 μl, i.n.) and the repeated administration (10 μg/30 μl, once per day for 5 days, i.n.) of MCH inhibited pain behaviors after CFA-induced inflammatory pain, suggesting a role of MCH as a potential analgesic agent. In this model, the analgesic effects of i.n. MCH lasted for at least 60 min after its administration, and the effects were equivalent to those of diclofenac (10 mg/kg, i.p.), an NSAID. The present study also compared the effects of MCH (10 μg/30 μl, i.n.) with or without the presence of a MCHR1 antagonist, TC-MCH 7c (10 mg/kg, i.p.), and found that the anti-allodynic effects of MCH totally disappeared following pretreatment with the MCHR1 antagonist. These results indicate that the effects of MCH were dependent on ligand–receptor interactions rather than due to unspecific effects.

Because the pharmacological treatment of neuropathic pain remains challenging^4,36^, the present study also examined the effects of MCH in the PSNL-induced neuropathic pain model. The repeated administration of MCH (10 μg/30 μl, once per day for 7 days, i.n.) produced a significant decrease in the mechanical allodynia induced by PSNL surgery and the degree of the anti-allodynic effect gradually increased over time.

Additionally, the present study aimed to characterize the endogenous analgesic mechanisms involved in MCH inhibition of CFA-induced inflammatory and PSNL-induced neuropathic pain using several receptor antagonists. The opioid receptor antagonist naltrexone blocked the anti-allodynic and analgesic effects of MCH in both pain models. Opioid receptors, the mu, kappa, and delta receptors, are widely distributed in the central and peripheral nervous systems. Opioids inhibit pain transmission mainly by interacting with these G protein-coupled receptors at presynaptic and postsynaptic sites in the dorsal horn of the spinal cord and by increasing descending inhibition from the brain and brainstem to the spinal cord^37^. The endogenous opioid system also inhibits nociceptive transmission in the ascendant pathway, activates the descending inhibitory pathway, and modifies the limbic system to inhibit the emotional perception of pain^38–40^. Some interactions between endogenous opioid and MCH systems have been proposed, including prevention of MCH-induced feeding by blockade of the kappa-, mu-, and delta-opioid receptors^41,42^, and antagonism of MCH-stimulated hedonic responses by kappa-, mu-, and delta-opioid receptor antagonists^43^. Further studies are needed to elucidate the roles of the endogenous opioid system in the modulation of pain by MCH.

Next, we demonstrated that the endocannabinoid system is also related to the analgesic effects of MCH, as the effects of i.n. MCH administration were markedly inhibited by a CB1R antagonist in both the inflammatory and neuropathic pain models. Several studies have suggested that there are interactions between the cannabinoid and MCH systems. For example, introduction of the endocannabinoid receptor ligand, 2-arachidonoyglycerol, into the rat lateral hypothalamus increases REM sleep and c-Fos expression in MCH neurons^44^ and combined treatment with CB1R and MCH1R antagonists synergistically induces anti-obesity effects in diet-induced obese mice^45^. The endocannabinoid system is an important regulator of the endogenous pain control system, which, along with the opioid system, plays an important role in the development and resolution of pain as well as in the affective components of pain^46,47^. The cannabinoid system also inhibits synaptic transmission and controls synaptic plasticity in pain pathways via activation of the G protein-coupled CB1 and CB2 cannabinoid receptors^28,48^. CB1Rs are widely distributed throughout the brain^48–50^, whereas CB2 receptors are most highly expressed in peripheral cells^48^. CBR activity inhibits ascending nociceptive transmission, activates the inhibitory descending pathway, and modifies the emotional aspects of pain^50,51^. These effects of the endocannabinoid system on the control of pain occur at the peripheral, spinal, and supraspinal levels^52^.

As current knowledge regarding the importance of the cannabinoid system in pain modulation has progressed, the mPFC has received an increasing amount of attention^53^. It is now known that the descending inhibitory pain system^36,54^ as well as higher centers of the brain, including the frontal lobe, anterior cingulate cortex, insula, and amygdala, are involved in the descending modulation of pain^54^. The mPFC is responsible for a variety of executive functions and is closely connected to limbic areas (e.g., amygdala) that play key roles in emotion^24,55,56^. Recent evidence has shown that pain-related neuroplastic changes in the mPFC–limbic network contribute to emotional responses and enhance pain sensitivity^57^ and that there are functional and structural abnormalities in the mPFC regions of patients with pain^58^. Additionally, rodent models of arthritis^59^ and neuropathic pain^60^ have observed reduced activity in output neurons in the mPFC. CB1Rs in the mPFC are thought to play an important role in the abnormal synaptic inhibition observed in pain models^61^; therefore, the restoration of mPFC outputs using CB1R agonists would be expected to alleviate pain^61,62^.

Based on the abovementioned findings, the present authors postulated that CB1Rs in the mPFC would be associated with the effects of MCH. MCH is effectively delivered and distributed to regions around the mPFC following the i.n. application of FITC-labeled MCH^7,18^, and it is known that MCH1Rs are expressed in this region as well^15^. In the present study, MCH treatment significantly restored reductions in the number of CB1Rs in the mPFC in the CFA group. Interestingly, the changes in CB1Rs in this region were negatively correlated with pain behaviors, which implies that the increased expression of CB1Rs may reflect decreases in pain. Furthermore, the MCH-induced increases in CB1Rs were blocked by a MCH1R antagonist, which indicates that MCH1Rs mediate the effects of MCH.

The present study also examined the activation of CB1Rs by assessing the number of cells that co-expressed c-Fos/CB1R. Although a decrease in activated CB1Rs was not evident, MCH treatment increased the activation of CB1Rs compared to the Control and CFA groups, and these changes were correlated with reductions in pain behaviors. It has been previously reported that microinjections of MCH into the dorsal raphe nucleus and basolateral amygdala induce depression-like behaviors^10,63^. However, contrary to previous findings, analyses of the TST and FST revealed that treatment with an anti-hyperalgesic dose of MCH for either 7 or 14 days decreased depression-like behaviors in the CFA and PSNL pain models. Thus, it is possible that changes in CB1Rs in the mPFC play important roles in the modulation of emotional deficits as well as in pain sensitivity.

Neuroimaging studies have shown that pain in humans is associated with activity in the mPFC and PAG^64^. These brain areas exhibit strong connectivity and form a network that allows for the top-down modulation of nociception^65^. Additionally, robust projections between the mPFC and PAG have been identified in rodents^66^, and the PAG is known to play an essential role in descending modulation through projections onto the rostral ventromedial medulla, which can exert either facilitating or inhibitory effects on the spinal cord^67–69^. Although MCH1Rs are not expressed in the mouse PAG^15^, it is possible that molecular changes in the mPFC (i.e., increased expression levels and the activation of CB1Rs) affect PAG activity and result in the modulation of pain transmission. In the present study, CB1R levels in the mPFC were highly correlated with those in the vlPAG and dlPAG. It is well known that CB1Rs activate the descending inhibitory pathway via the inhibition of GABA release in the PAG^48,70^. The immunofluorescence analyses performed in the present study demonstrated that the expression levels of CB1Rs within the vlPAG and dlPAG were lowered in the inflammatory pain model, which is consistent with previous findings^71–73^. In contrast, the decreases in the expressions of CB1Rs were attenuated following i.n. MCH treatment but were inhibited by pretreatment with a MCH1R antagonist. Furthermore, MCH treatment enhanced the activation of CB1Rs in these areas. Consequently, it can be postulated that the i.n. administration of MCH modulated higher centers of the brain, including the mPFC, which subsequently induced changes in the PAG and resulted in the activation of the descending inhibitory pathway. However, further studies are required to gain a better understanding of the contribution of the cannabinoid system to the analgesic effects of MCH.

In the present study, MCH was delivered via i.n. administration, which allowed the peptides to rapidly enter the brain and directly bypass the blood–brain barrier^74–76^. The intranasally administered FITC-labeled MCH (10 μg/30 μl) was distributed to various brain regions, including the PFC, nucleus accumbens, and hippocampus, within 30 min of administration (data not shown). This finding is consistent with those of previous studies showing that peptides delivered via i.n. administration are detected at maximal levels after 30 min and can be detected after 6 h^76,77^. Although changes in CB1R levels were observed in the mPFC, vlPAG, and dlPAG following the administration of i.n. MCH, other brain regions (including the spinal cord, striatum, nucleus accumbens, and amygdala) may also contribute to the blockade of allodynic and hyperalgesic abnormalities associated with inflammatory and neuropathic pain.

Intranasally administered MCH can spread to various parts of the brain as well as to the blood stream. In addition to its localization in the CNS, MCH1Rs are also found in peripheral tissues, such as the thyroid, kidney, adipose tissue, lung, testes, and tongue^78,79^, where they perform various physiological functions. Although the peripheral actions of MCH are not yet well understood relative to its functions in the CNS, lower levels of intestinal inflammation^80^ and intestinal tumorigenesis^81^ and an increased mortality against intestinal pathogens^82^ are evident in MCH KO mice, which suggests that the peripheral functions of MCH should be considered^82^.

The present study also investigated whether the administration of MCH (i.n.) for 5 consecutive days would induce intestinal inflammation by measuring macroscopic damage in terms of hyperemia, changes in the thickness of the colon wall, and the extent of ulceration^80^. There were no abnormal signs of inflammation in the intestines of animals in the CFA-induced pain model (Supplementary Fig. 2). The present study also examined the effects of repeated MCH treatments on body weight and food intake in healthy mice for 3 weeks because MCH was found to increase body weight and food intake in previous studies^29,45^. However, there were no significant increases in body weight or food intake in MCH-treated mice compared with controls (data not shown). These findings suggest that i.n. MCH treatment could be used as an analgesic agent without side effects, such as weight gain. In any event, further long-term observation will be necessary to monitor the occurrence of possible adverse events.

In conclusion, the present data demonstrated potential analgesic effects of MCH via i.n. administration on both inflammatory and neuropathic pain, thus suggesting new potential therapeutic applications of this peptide in addition to its previously described effects.

## Methods

### Animals

The present study included 8-week-old male C57BL/6 mice weighing 24–26 g (Samtaco; Seoul, Korea). pMCH^−/−^ mice with a C57BL/6 background^83^ were obtained from Jackson Laboratory (Bar Harbor, ME, USA), and littermates were generated by breeding heterozygous mutants. All animals were housed at 24 ± 2 °C under a 12-hour light/dark cycle (lights on from 08:00 to 20:00) with free access to food and water for at least 1 week prior to commencement of the experiments. All animals were randomly assigned to each group. All experiments were performed in accordance with the institutional guidelines and regulations for the care and use of laboratory animals, and all experiments were approved by Kyung Hee University Animal Care Committee for Animal Welfare (KHUASP(SE)-11-022), Seoul, Korea. The authors made every effort to minimize the number of animals used and their suffering.

### Induction of inflammatory pain using CFA

In the present study, a CFA oil suspension diluted with saline (1:1) was used to induce chronic inflammation pain. All mice were anesthetized with ether prior to injection and then received an intraplantar (i.pl.) injection of a CFA (100 μl, Sigma; St. Louis, MO, USA) emulsion solution into both hind paws bilaterally. The Control group received an identical injection of an equal amount of saline^2,84^.

### Induction of neuropathic pain by PSNL

A PSNL-induced pain model developed by Malmberg and Basbaum^85^ was employed in the present study. All mice were anesthetized with 2% Zoletil (100 μl, i.p.; Virbac S.A.; Carros, France) and Rompun (100 μl, i.p.; Bayer; Seoul, Korea), and the left thigh was then shaved so the sciatic nerve was exposed. The dorsal one-third to one-half of the nerve was loosely ligated with 8-0 silk (AILEE; Busan, Korea), and the wound was closed. In the Sham group, the nerve was exposed without ligation. Animals were assigned to each group by random allocation, and baseline testing was conducted on Day 7 after surgery.

### I.n. administration of MCH

I.n. administration is a noninvasive method used to bypass the blood–brain barrier that allows for the delivery of drugs from the nasal cavity to the brain^86^. Although microinjections have been widely used for the brain-specific delivery of MCH^10,30^, this surgical method is quite invasive and requires the anesthetization of experimental animals. Therefore, the i.n. administration procedure was performed as an alternative method of delivering MCH into the brain, as described previously^75^. Briefly, MCH (3806, Tocris Biosciences; Bristol, UK) was dissolved in saline, and the MCH solution (5, 10, 15, 20, or 25 μg/30 μl) was then dropped onto the noses of the mice, which caused them to inhale the solution. The mice in the control/sham and pain model groups inhaled an equal amount of i.n. saline to approximate equal levels of stress.

In the CFA group, either a single administration of MCH (10 μg/30 μl) on the 4^th^ day after the CFA injection or repeated treatment with MCH (10 μg/30 μl) for 5 consecutive days from the 4^th^ day after CFA injection was employed. Alternatively, diclofenac (10 mg/kg, i.p.; Sigma) was dissolved in saline and administered 4 days after the CFA injection. In the PSNL-induced pain model, chronic i.n. MCH was administered for 7 consecutive days from Day 7 after the PSNL surgery. All treatments were performed at the same time every day to control for circadian differences.

### Mechanical allodynia test

Mechanical allodynia in both hind paws was assessed using electronic von Frey filaments (IITC; Woodland Hills, CA, USA) in both the CFA and PSNL-induced pain models. Prior to beginning the present experiment, all mice were habituated in a clear 8 × 10 × 10-cm acrylic box with a gridded floor to allow for adaptation to the experimental environment. Adaptation was performed for 1 h each day for 2 days, and baseline behaviors were assessed at least 1 day prior to the saline/CFA injection and sham/PSNL surgery. Subsequently, all mice were returned to the same clear box, and the mechanical thresholds were assessed by applying the filament to each mouse 10 times at a force of 0.6 g to the plantar surface of each hind paw with a 5-s gap between each application. The frequency of positive responses among the 10 applications was counted^2^, and mechanical allodynia was defined as an increase in the withdrawal frequency in response to non-painful stimulation. The values of the bilateral hind paws in the CFA-evoked pain model were averaged. Additionally, to determine whether there was any difference in the induction time required to reach a peak level of pain, the withdrawal frequency was converted to a relative value of the peak level of pain, and the data were then compared. The peak level of pain was defined as a difference of >7 points in the withdrawal frequency, and the relative allodynic response was then calculated (% of the peak level of pain).

### Thermal hyperalgesia test

Thermal hyperalgesia was measured using the hotplate test (Bioseb; Pinellas Park, FL, USA) in the CFA-induced pain model. Mice were placed on a hot plate maintained at 52.5 °C and the times to lick any side of the hind paw or to jumping were analyzed (latency time). The time on the plate was recorded for a maximum of 40 s to avoid tissue damage and the values of two trials were averaged. The baseline measurement was conducted 1 day prior to saline or CFA administration (i.pl.). The test measurements were then performed 1 h prior to the MCH or saline i.n. treatments (0 min), which were measured 30 and 60 min after the MCH or saline i.n. administrations, respectively.

### Depression-like behavior tests

Depression-like behaviors were assessed with the TST or FST and all behavioral data were recorded using a video camera (LCS-X30, SONY; Tokyo, Japan). For the TST, mice were suspended by their tails 50 cm above the floor using labeling tape and immobility time was recorded for 6 min. For the FST, mice were placed in a cylinder (10 cm × 25 cm; diameter × height) filled with water at a temperature of 27 °C for 6 min. Immobility time was monitored during the last 4 min of the 6-min test and defined as the absence of escape-oriented behavior. Immediately after the trial, mice were placed under a heating towel to dry.

### Effects of MCH1R, opioid, cannabinoid, adrenergic, and/or serotonergic receptor antagonists on MCH activity

Pain is modulated by several pathways including the opioid, cannabinoid, adrenergic, and serotonergic systems. In the present study, antagonist challenges were applied to determine the involvement of these specific analgesic mechanisms in the effects of MCH. An opioid receptor antagonist (naltrexone, 5 mg/kg; Sigma), α1 adrenoreceptor antagonist (prazosin hydrochloride, 1 mg/kg; Sigma), α2 adrenoreceptor antagonist (yohimbine hydrochloride, 1 mg/kg; Sigma), and 5-HT1/2 receptor antagonist (cyproheptadine hydrochloride, 1 mg/kg; Sigma) were dissolved in saline^87^. While a CB1R antagonist (AM251, 4 mg/kg; Sigma) was dissolved in a solution of Tween-80, ethanol, and saline (1:1:18)^88,89^. Additionally, to investigate whether the effects of MCH were mediated via MCH1R, a MCH1R antagonist, TC-MCH 7c (10 mg/kg; Tocris Biosciences) was dissolved in 1% dimethyl sulfoxide (DMSO; Sigma)^90^ prior to its administration. All receptor antagonists were intraperitoneally injected 30 min prior to MCH treatment.

### Immunofluorescence analysis

All animals were slightly anesthetized with dimethyl ether (Sigma) inhalation anesthesia transcardially perfused with 1 × phosphate buffered saline (PBS, Biomedic; Seoul, Korea) followed by ice-cold formalin (10%; Sigma). The brains were removed, post-fixed, and cryoprotected overnight in sucrose (30% w/v in 1 × PBS) before being sectioned on a freezing microtome (CM 1850, Leica; Nussloch, Germany). For the double immunofluorescence procedure, coronal sections that included the PFC and PAG (40 μm thick) were selected for c-Fos/CB1R staining (two sections per mouse; *n* = 6/group). The selected sections were washed three times and then incubated in 5% bovine serum albumin (BSA, Merck Millipore; Darmstadt, Germany) and 0.2% Triton X-100 (Sigma) in 1× PBS (PBST) for 1 h. Primary antibodies raised against NeuN (mouse, 1:500, MAB377; Chemicon International, Inc.; Temecula, California), c-Fos (rabbit, 1:200, SC-52; Santa Cruz Biotechnology; Santa Cruz, CA, USA), and CB1R (goat, 1:500, SC-10066; Santa Cruz Biotechnology) were diluted in 1× PBST supplemented with 0.1% BSA. The plates were wrapped in foil to block light and then stored at 4 °C for 72 h. Next, the tissue sections were washed in PBST and sequentially incubated for 1 h with a mixture of Alexa 488 conjugated donkey anti-mouse secondary antibody (1:1000; Thermo Fisher Scientific; Fremont, CA, USA), Alexa 488 conjugated donkey anti-rabbit secondary antibody (1:100; Thermo Fisher Scientific), and Alexa 594 conjugated donkey anti-goat secondary antibody (1:500; Thermo Fisher Scientific). For primary antibody control purposes, brain sections were performed with 1× PBST instead of primary antibody. Additionally, to further check the specificity, the primary CB1R antibody was preabsorbed overnight at 4 °C with its CB1R peptide (1:10, ab50542; Abcam, Cambridge, UK) before incubation on the brain sections.

After the staining procedure was completed, the tissues were mounted on gelatinized slides and coverslipped. The numbers of c-Fos and CB1R double-positive cells within the mPFC, vlPAG, and dlPAG were counted three times by a researcher blind to each group. Cell counting was performed using a square grid (200 μm × 200 μm) in each brain region and the mean counts obtained were defined as the numbers of c-Fos and CB1R positive cells. All images were obtained using a confocal fluorescence microscope (BX53; Olympus Corporation; Tokyo, Japan). Additionally, to certify that the signals were trustworthy, no primary antibody controls were used.

### Distribution of FITC-labelled MCH after i.n. administration

Thirty minutes after the i.n. administration of either MCH-FITC (10 μg/30 μl, GL Biochem; Shanghai, China) or saline (30 μl), the slightly anesthetized mice were transcardially perfused with PBS followed by ice-cold 10% formalin. The brains were immediately removed and stored at −80 °C for 1 h. Next, coronal sections (40 μm thick) of the brain were cut to encompass the entire brain. Coverslips were mounted using mounting medium containing DAPI (H-1200, Vector Laboratories Inc.; Burlingame, CA, USA) and all images were obtained using a confocal fluorescence microscope (BX53, Olympus Corporation).

### Macroscopic examination

Possible side effects of MCH were evaluated by a macroscopic examination of intestinal status after treatment with MCH (10 μg/30 μl) for 5 consecutive days. The small and large intestines of the anesthetized mice were immediately removed after sacrifice and the degree of thickness of the colon wall, hyperemia, and the extent of ulceration were assessed.

### Statistical analysis

All statistical parameters were calculated using GraphPad Prism 5.0 software (GraphPad Software; San Diego, CA, USA). Unpaired two-tailed t-test was used for comparing the different of pain responses between MCH^+/+^ and MCH^−/−^ mice. Group comparisons of dose–response data based on thermal pain threshold, depression-like behaviors based on the TST and FST, and immunohistochemical data were performed with one-way analysis of variance (ANOVA) tests followed by Newman–Keuls tests. Analyses of mechanical and thermal pain at various timepoints were performed with two-way repeated measures ANOVAs and Bonferroni post hoc tests for pairwise multiple comparisons. Spearman rank correlation coefficient tests were performed to analyze whether the changes in CB1Rs and their activation in the mPFC and PAG were correlated with pain behaviors. all data are expressed as a mean ± standard error of the mean (SEM). For all analyses, *p* < 0.05 was considered to indicate statistical significance.

## Electronic supplementary material

Supplementary figure

## References

[1] Angst MS (2012). Pain sensitivity and opioid analgesia: a pharmacogenomic twin study. Pain.

[2] Park JY (2014). From peripheral to central: the role of ERK signaling pathway in acupuncture analgesia. J Pain.

[3] Benyamin R (2008). Opioid complications and side effects. Pain Physician.

[4] Moulin DE (2007). Pharmacological management of chronic neuropathic pain - consensus statement and guidelines from the Canadian Pain Society. Pain Res Manag.

[5] Andreev N, Urban L, Dray A (1994). Opioids suppress spontaneous activity of polymodal nociceptors in rat paw skin induced by ultraviolet irradiation. Neuroscience.

[6] Fisher K, Coderre TJ, Hagen NA (2000). Targeting the N-methyl-D-aspartate receptor for chronic pain management. Preclinical animal studies, recent clinical experience and future research directions. J Pain Symptom Manage.

[7] Bittencourt JC (1992). The melanin-concentrating hormone system of the rat brain: an immuno- and hybridization histochemical characterization. J Comp Neurol.

[8] Torterolo P, Sampogna S, Morales FR, Chase MH (2006). MCH-containing neurons in the hypothalamus of the cat: searching for a role in the control of sleep and wakefulness. Brain Res.

[9] Torterolo P, Sampogna S, Chase MH (2009). MCHergic projections to the nucleus pontis oralis participate in the control of active (REM) sleep. Brain Res.

[10] Kim TK (2015). Antidepressant effects of exercise are produced via suppression of hypocretin/orexin and melanin-concentrating hormone in the basolateral amygdala. Neurobiol Dis.

[11] Garcia-Fuster MJ (2012). The melanin-concentrating hormone (MCH) system in an animal model of depression-like behavior. Eur Neuropsychopharmacol.

[12] Pissios P (2008). Dysregulation of the mesolimbic dopamine system and reward in MCH−/− mice. Biol Psychiatry.

[13] DiLeone RJ, Georgescu D, Nestler EJ (2003). Lateral hypothalamic neuropeptides in reward and drug addiction. Life Sci.

[14] Saito Y, Nagasaki H (2008). The melanin-concentrating hormone system and its physiological functions. Results Probl Cell Differ.

[15] Chee MJ, Pissios P, Maratos-Flier E (2013). Neurochemical characterization of neurons expressing melanin-concentrating hormone receptor 1 in the mouse hypothalamus. J Comp Neurol.

[16] Rodriguez M (2001). Cloning and molecular characterization of the novel human melanin-concentrating hormone receptor MCH2. Mol Pharmacol.

[17] Tan CP (2002). Melanin-concentrating hormone receptor subtypes 1 and 2: species-specific gene expression. Genomics.

[18] Saito Y, Cheng M, Leslie FM, Civelli O (2001). Expression of the melanin-concentrating hormone (MCH) receptor mRNA in the rat brain. J Comp Neurol.

[19] Erthal V, Nohama P (2015). Treatment for neuropathic pain and chronic inflammation using LASER in animal models. Conf Proc IEEE Eng Med Biol Soc.

[20] Wang XQ (2015). Differential roles of hippocampal glutamatergic receptors in neuropathic anxiety-like behavior after partial sciatic nerve ligation in rats. BMC Neurosci.

[21] Bushnell MC (2015). Effect of environment on the long-term consequences of chronic pain. Pain.

[22] Le AM, Lee M, Su C, Zou A, Wang J (2014). AMPAkines have novel analgesic properties in rat models of persistent neuropathic and inflammatory pain. Anesthesiology.

[23] Miller LR, Cano A (2009). Comorbid chronic pain and depression: who is at risk?. J Pain.

[24] Morgan MA, LeDoux JE (1995). Differential contribution of dorsal and ventral medial prefrontal cortex to the acquisition and extinction of conditioned fear in rats. Behav Neurosci.

[25] Millecamps M (2007). D-cycloserine reduces neuropathic pain behavior through limbic NMDA-mediated circuitry. Pain.

[26] Tajerian M (2013). Peripheral nerve injury is associated with chronic, reversible changes in global DNA methylation in the mouse prefrontal cortex. PLoS One.

[27] Galdino G (2014). Acute resistance exercise induces antinociception by activation of the endocannabinoid system in rats. Anesth Analg.

[28] Galdino G (2014). The endocannabinoid system mediates aerobic exercise-induced antinociception in rats. Neuropharmacology.

[29] Noseda R, Kainz V, Borsook D, Burstein R (2014). Neurochemical pathways that converge on thalamic trigeminovascular neurons: potential substrate for modulation of migraine by sleep, food intake, stress and anxiety. PLoS One.

[30] Devera A (2015). Melanin-concentrating hormone (MCH) modulates the activity of dorsal raphe neurons. Brain Res.

[31] Torterolo P (2015). Melanin-Concentrating Hormone (MCH): Role in REM Sleep and Depression. Front Neurosci.

[32] Blouin AM (2013). Human hypocretin and melanin-concentrating hormone levels are linked to emotion and social interaction. Nat Commun.

[33] Nahon JL, Presse F, Bittencourt JC, Sawchenko PE, Vale W (1989). The rat melanin-concentrating hormone messenger ribonucleic acid encodes multiple putative neuropeptides coexpressed in the dorsolateral hypothalamus. Endocrinology.

[34] Toumaniantz G, Bittencourt JC, Nahon JL (1996). The rat melanin-concentrating hormone gene encodes an additional putative protein in a different reading frame. Endocrinology.

[35] Borsu L, Presse F, Nahon JL (2000). The AROM gene, spliced mRNAs encoding new DNA/RNA-binding proteins are transcribed from the opposite strand of the melanin-concentrating hormone gene in mammals. J Biol Chem.

[36] Kwon M, Altin M, Duenas H, Alev L (2014). The role of descending inhibitory pathways on chronic pain modulation and clinical implications. Pain Pract.

[37] Wall PD, McMahon SB, Koltzenburg M (2006). Wall and Melzack’s textbook of pain.

[38] Gebhart GF (2004). Descending modulation of pain. Neurosci Biobehav Rev.

[39] Negrete R, Garcia Gutierrez MS, Manzanares J, Maldonado R (2016). Involvement of the dynorphin/KOR system on the nociceptive, emotional and cognitive manifestations of joint pain in mice. Neuropharmacology.

[40] Kieffer BL, Gaveriaux-Ruff C (2002). Exploring the opioid system by gene knockout. Prog Neurobiol.

[41] Arora S (2006). & Anubhuti. Role of neuropeptides in appetite regulation and obesity–a review. Neuropeptides.

[42] Baile CA, McLaughlin CL, Della-Fera MA (1986). Role of cholecystokinin and opioid peptides in control of food intake. Physiol Rev.

[43] Lopez CA (2011). Involvement of the opioid system in the orexigenic and hedonic effects of melanin-concentrating hormone. Am J Physiol Regul Integr Comp Physiol.

[44] Perez-Morales, M. *et al*. 2-AG into the lateral hypothalamus increases REM sleep and cFos expression in melanin concentrating hormone neurons in rats. *Pharmacol Biochem Behav* **108**, 1–7 (2013). 10.1016/j.pbb.2013.04.006 23603032

[45] Verty AN, Lockie SH, Stefanidis A, Oldfield BJ (2013). Anti-obesity effects of the combined administration of CB1 receptor antagonist rimonabant and melanin-concentrating hormone antagonist SNAP-94847 in diet-induced obese mice. Int J Obes (Lond).

[46] Woodhams SG, Sagar DR, Burston JJ, Chapman V (2015). The role of the endocannabinoid system in pain. Handb Exp Pharmacol.

[47] Abrams DI, Couey P, Shade SB, Kelly ME, Benowitz NL (2011). Cannabinoid-opioid interaction in chronic pain. Clin Pharmacol Ther.

[48] Ulugol A (2014). The endocannabinoid system as a potential therapeutic target for pain modulation. Balkan Med J.

[49] Moreira FA, Wotjak CT (2010). Cannabinoids and anxiety. Curr Top Behav Neurosci.

[50] Huang WJ, Chen WW, Zhang X (2016). Endocannabinoid system: Role in depression, reward and pain control (Review). Mol Med Rep.

[51] Hama A, Sagen J (2007). Antinociceptive effect of cannabinoid agonist WIN 55,212-2 in rats with a spinal cord injury. Exp Neurol.

[52] Hama A, Sagen J (2011). Activation of spinal and supraspinal cannabinoid-1 receptors leads to antinociception in a rat model of neuropathic spinal cord injury pain. Brain Res.

[53] Woodhams SG, Chapman V, Finn DP, Hohmann AG, Neugebauer V (2017). The cannabinoid system and pain. Neuropharmacology.

[54] Tracey I, Mantyh PW (2007). The cerebral signature for pain perception and its modulation. Neuron.

[55] Viviani R (2014). Neural correlates of emotion regulation in the ventral prefrontal cortex and the encoding of subjective value and economic utility. Front Psychiatry.

[56] Chang, C. H. & Ho, T. W. Inhibitory modulation of medial prefrontal cortical activation on lateral orbitofrontal cortex-amygdala information flow. *J Physiol* **595**(17), 6065–6076 (2017). 10.1113/JP274568 PMC5577524 28678402

[57] Neugebauer V (2015). Amygdala pain mechanisms. Handb Exp Pharmacol.

[58] Apkarian AV (2004). Chronic back pain is associated with decreased prefrontal and thalamic gray matter density. J Neurosci.

[59] Ji G, Neugebauer V (2011). Pain-related deactivation of medial prefrontal cortical neurons involves mGluR1 and GABA(A) receptors. J Neurophysiol.

[60] Zhang Z (2015). Role of Prelimbic GABAergic Circuits in Sensory and Emotional Aspects of Neuropathic Pain. Cell Rep.

[61] Kiritoshi T, Ji G, Neugebauer V (2016). Rescue of Impaired mGluR5-Driven Endocannabinoid Signaling Restores Prefrontal Cortical Output to Inhibit Pain in Arthritic Rats. J Neurosci.

[62] Kiritoshi T (2013). Modulation of pyramidal cell output in the medial prefrontal cortex by mGluR5 interacting with CB1. Neuropharmacology.

[63] Lagos P, Urbanavicius J, Scorza MC, Miraballes R, Torterolo P (2011). Depressive-like profile induced by MCH microinjections into the dorsal raphe nucleus evaluated in the forced swim test. Behav Brain Res.

[64] Yu R (2014). Disrupted functional connectivity of the periaqueductal gray in chronic low back pain. Neuroimage Clin.

[65] Klug S (2011). Dysfunctional pain modulation in somatoform pain disorder patients. Eur Arch Psychiatry Clin Neurosci.

[66] Floyd NS, Price JL, Ferry AT, Keay KA, Bandler R (2000). Orbitomedial prefrontal cortical projections to distinct longitudinal columns of the periaqueductal gray in the rat. J Comp Neurol.

[67] Behbehani MM (1995). Functional characteristics of the midbrain periaqueductal gray. Prog Neurobiol.

[68] Sandkuhler J, Gebhart GF (1984). Relative contributions of the nucleus raphe magnus and adjacent medullary reticular formation to the inhibition by stimulation in the periaqueductal gray of a spinal nociceptive reflex in the pentobarbital-anesthetized rat. Brain Res.

[69] Fields HL, Malick A, Burstein R (1995). Dorsal horn projection targets of ON and OFF cells in the rostral ventromedial medulla. J Neurophysiol.

[70] Ho YC (2011). Activation of orexin 1 receptors in the periaqueductal gray of male rats leads to antinociception via retrograde endocannabinoid (2-arachidonoylglycerol)-induced disinhibition. J Neurosci.

[71] Knerlich-Lukoschus F (2011). Spinal cord injuries induce changes in CB1 cannabinoid receptor and C-C chemokine expression in brain areas underlying circuitry of chronic pain conditions. J Neurotrauma.

[72] Palazzo E (2012). Changes in cannabinoid receptor subtype 1 activity and interaction with metabotropic glutamate subtype 5 receptors in the periaqueductal gray-rostral ventromedial medulla pathway in a rodent neuropathic pain model. CNS Neurol Disord Drug Targets.

[73] Li MH, Suchland KL, Ingram SL (2017). Compensatory Activation of Cannabinoid CB2 Receptor Inhibition of GABA Release in the Rostral Ventromedial Medulla in Inflammatory Pain. J Neurosci.

[74] Serova LI, Laukova M, Alaluf LG, Sabban EL (2013). Intranasal infusion of melanocortin receptor four (MC4R) antagonist to rats ameliorates development of depression and anxiety related symptoms induced by single prolonged stress. Behav Brain Res.

[75] Kim SN (2011). Phosphatidylinositol 3-kinase/Akt signaling pathway mediates acupuncture-induced dopaminergic neuron protection and motor function improvement in a mouse model of Parkinson’s disease. Int J Neurosci.

[76] Thorne RG, Pronk GJ, Padmanabhan V, Frey WH (2004). Delivery of insulin-like growth factor-I to the rat brain and spinal cord along olfactory and trigeminal pathways following intranasal administration. Neuroscience.

[77] Ionescu IA (2012). Intranasally administered neuropeptide S (NPS) exerts anxiolytic effects following internalization into NPS receptor-expressing neurons. Neuropsychopharmacology.

[78] Chung S (2012). Disruption of the melanin-concentrating hormone receptor 1 (MCH1R) affects thyroid function. Endocrinology.

[79] Schlumberger SE, Talke-Messerer C, Zumsteg U, Eberle AN (2002). Expression of receptors for melanin-concentrating hormone (MCH) in different tissues and cell lines. J Recept Signal Transduct Res.

[80] Kokkotou E (2008). Melanin-concentrating hormone as a mediator of intestinal inflammation. Proc Natl Acad Sci USA.

[81] Nagel JM (2012). Reduced intestinal tumorigenesis in APCmin mice lacking melanin-concentrating hormone. PLoS One.

[82] Karagiannis AK (2013). Increased susceptibility of melanin-concentrating hormone-deficient mice to infection with Salmonella enterica serovar Typhimurium. Infect Immun.

[83] Conductier G (2013). Melanin-concentrating hormone regulates beat frequency of ependymal cilia and ventricular volume. Nat Neurosci.

[84] Malin SA (2008). Thermal nociception and TRPV1 function are attenuated in mice lacking the nucleotide receptor P2Y2. Pain.

[85] Malmberg AB, Basbaum AI (1998). Partial sciatic nerve injury in the mouse as a model of neuropathic pain: behavioral and neuroanatomical correlates. Pain.

[86] Gozes I (2001). Neuroprotective peptide drug delivery and development: potential new therapeutics. Trends Neurosci.

[87] Kaygisiz B, Kilic FS, Senguleroglu N, Baydemir C, Erol K (2015). The antinociceptive effect and mechanisms of action of pregabalin in mice. Pharmacol Rep.

[88] Godin AM (2015). Activities of 2-phthalimidethyl nitrate and 2-phthalimidethanol in the models of nociceptive response and edema induced by formaldehyde in mice and preliminary investigation of the underlying mechanisms. Eur J Pharmacol.

[89] Takeuchi Y, Takasu K, Ono H, Tanabe M (2007). Pregabalin, S-(+)-3-isobutylgaba, activates the descending noradrenergic system to alleviate neuropathic pain in the mouse partial sciatic nerve ligation model. Neuropharmacology.

[90] Park, J. Y. *et al*. Novel Neuroprotective Effects of Melanin-Concentrating Hormone in Parkinson’s Disease. *Mol Neurobiol* **54**(10), 7706–7721 (2017). 10.1007/s12035-016-0258-8 27844281

