# Supplementary material for: Novel analgesic effects of melanin-concentrating hormone on persistent neuropathic and inflammatory pain in mice
Source: Sci Rep. 2018 Jan 15;8:707. doi: 10.1038/s41598-018-19145-z (PMC5768747; doi:10.1038/s41598-018-19145-z)
Supplement: Supplementary file 1 — Supplementary figure [file 41598_2018_19145_MOESM1_ESM.pdf]

Novel analgesic effects of melanin-concentrating hormone on persistent neuropathic and inflammatory pain in mice

Jae-Hwan Jang[1,2,3], Ji-Yeun Park[4], Ju-Young Oh[1,2,3], Sun-Jeong Bae[1], Hyunchul Jang[5], Songhee Jeon[6*], Jongpil Kim[7*] & Hi-Joon Park[1,2,3*]

Supplementary Information

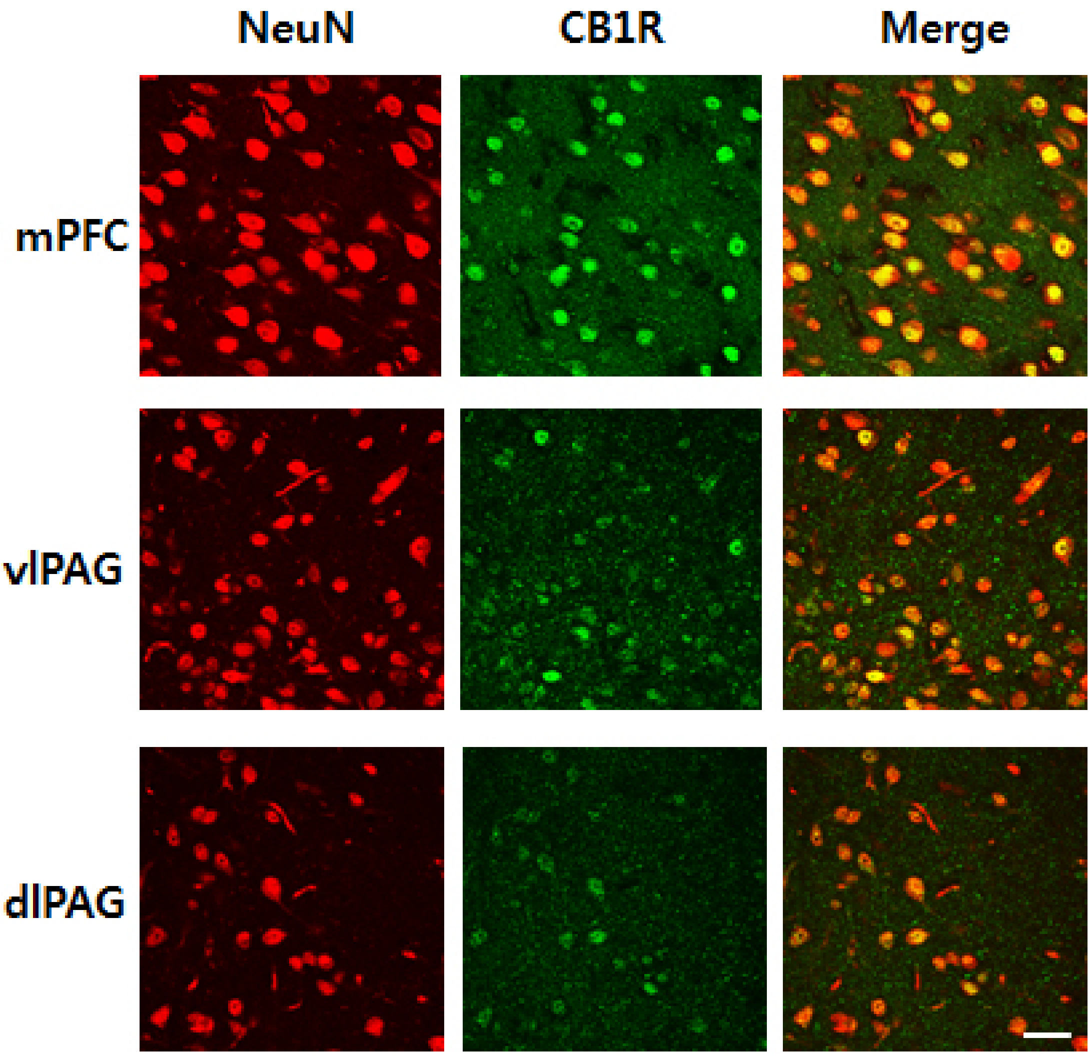

Supplementary Figure 1. The expression of CB1R in neurons in the mPFC, vl- and dlPAG. Histological examination of tissue sections from the mPFC, vl- and dlPAG of mice using immunofluorescence staining showing the expression of CB1R (green), NeuN (red) and co-expression of CB1R and NeuN (orange). Scale bar: 30 μm.

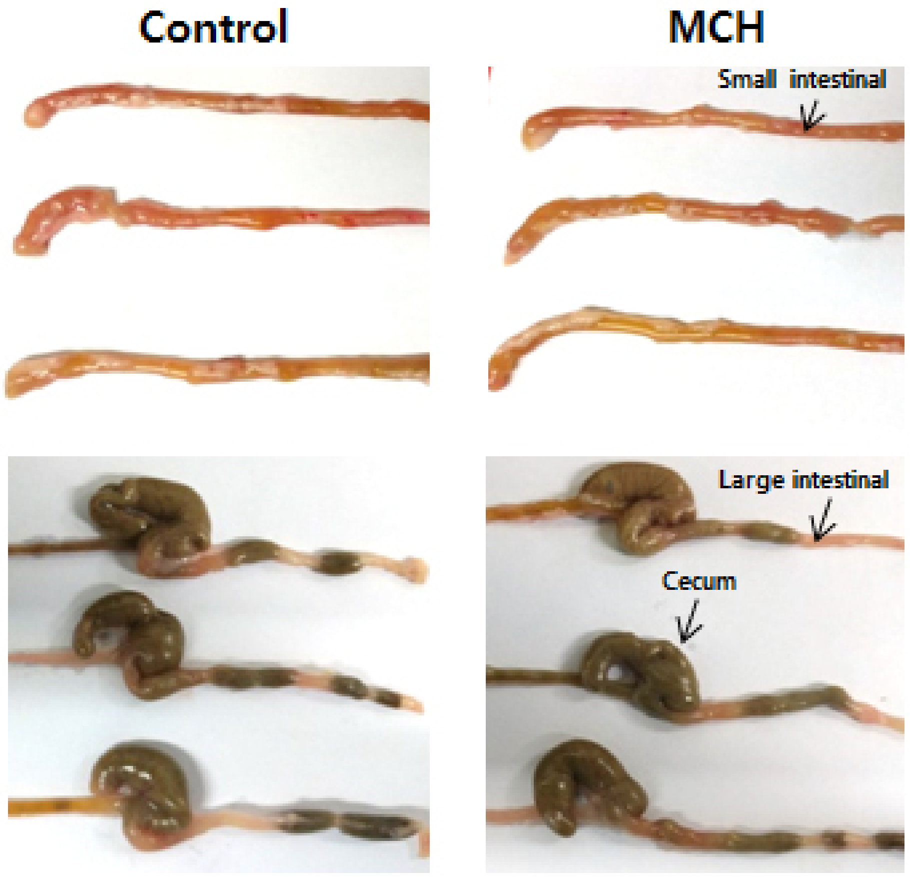

Supplementary Figure 2. The intestinal status after i.n administration MCH in the mice. Macroscopic examination of intestinal tissue from the mice after administration of MCH (10 μg/30 μl) for 5 consecutive days. There were no signs of inflammation after MCH treatments.

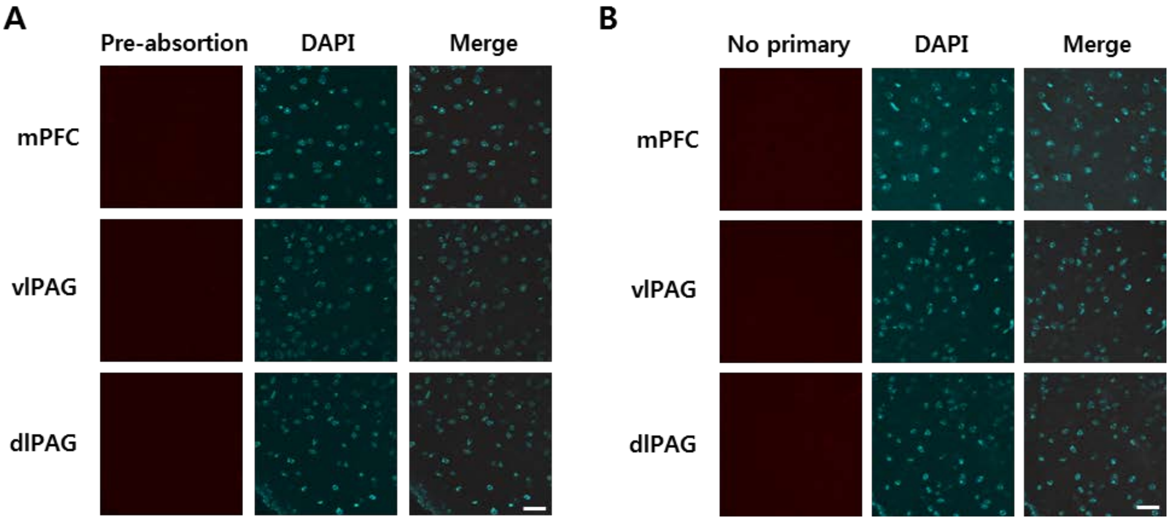

Supplementary Figure 3. Control Images to show the specificity of the CB1R immunohistochemistry in mPFC, vl- and dlPAG. (A) shows that the immunoreactivity of CB1R totally disappeared when the tissues were processed for CB1R antibody preabsorbed with CB1R antigen. (B) shows that the immunoreactivity of CB1R was not found when the tissues were incubated with the antibody diluent alone without primary CB1R antibody. CB1R (red) and DAPI (blue). Scale bar: 30 µm.
